# Convergent synthesis of diversified reversible network leads to liquid metal-containing conductive hydrogel adhesives

**DOI:** 10.1038/s41467-021-22675-2

**Published:** 2021-04-23

**Authors:** Yong Xu, Rebecca Rothe, Dagmar Voigt, Sandra Hauser, Meiying Cui, Takuya Miyagawa, Michelle Patino Gaillez, Thomas Kurth, Martin Bornhäuser, Jens Pietzsch, Yixin Zhang

**Affiliations:** 1 Technische Universität Dresden, B CUBE Center for Molecular Bioengineering, Dresden, Germany; 2 Helmholtz-Zentrum Dresden-Rossendorf (HZDR), Institute of Radiopharmaceutical Cancer Research Department of Radiopharmaceutical and Chemical Biology, Dresden, Germany; 3 Technische Universität Dresden, School of Science, Faculty of Chemistry and Food Chemistry, Dresden, Germany; 4 Technische Universität Dresden, Institute for Botany, Faculty of Biology, Dresden, Germany; 5 Technische Universität Dresden, Center for Molecular and Cellular Bioengineering (CMCB), Technology Platform, EM Facilty, Dresden, Germany; 6 Center for Regenerative Therapies Dresden (CRTD), Technische Universität Dresden, Dresden, Germany; 7 University Hospital Carl Gustav Carus der Technischen Universität Dresden, Medizinische Klinik und Poliklinik I, Dresden, Germany; 8 Cluster of Excellence Physics of Life, Technische Universität Dresden, Dresden, Germany

**Keywords:** Bioinspired materials, Biomedical materials, Electronic properties and materials, Molecular self-assembly

## Abstract

Many features of extracellular matrices, e.g., self-healing, adhesiveness, viscoelasticity, and conductivity, are associated with the intricate networks composed of many different covalent and non-covalent chemical bonds. Whereas a reductionism approach would have the limitation to fully recapitulate various biological properties with simple chemical structures, mimicking such sophisticated networks by incorporating many different functional groups in a macromolecular system is synthetically challenging. Herein, we propose a strategy of convergent synthesis of complex polymer networks to produce biomimetic electroconductive liquid metal hydrogels. Four precursors could be individually synthesized in one to two reaction steps and characterized, then assembled to form hydrogel adhesives. The convergent synthesis allows us to combine materials of different natures to generate matrices with high adhesive strength, enhanced electroconductivity, good cytocompatibility in vitro and high biocompatibility in vivo. The reversible networks exhibit self-healing and shear-thinning properties, thus allowing for 3D printing and minimally invasive injection for in vivo experiments.

## Abstract (web summary)

The need for multifunctional materials for tissue engineering applications requires the development of multicomponent systems. Here, the authors report on the creation of a liquid metal-containing hydrogel with multiple covalent and noncovalent bonds to produce a tailorable, biocompatible biomaterial.

## Introduction

Whereas synthetic materials are developed and optimized for certain utilities, biological materials have developed through evolution to possess multifunctionality, which can be as diverse and versatile as the phenomena of life. For example, extracellular matrices (ECM) are biopolymers with a long list of functions, including scaffolds with desired mechanical features, adhesive properties for connecting cells and tissues, semi-conductive environment required for electric signaling, limited diffusion of signaling molecules to generate gradients, etc.^1–4^. A bottom-up approach to construct synthetic systems to mimic the multifunctional ECMs will help to understand the molecular basis of biological ECMs, as well as to develop multi-functional, tunable materials for biotechnological, diagnostic, and therapeutic applications^5,6^.

Among functional bio-inspired materials, conductive hydrogels have gained increasing interest for many biomedical applications, for their great potentials to couple detection and stimulation of biological processes with electric and information technologies^7–9^. Adhesion and injectability are also highly desired features to facilitate clinical and surgical utility^10,11^. To design such materials, a bio-inspired approach could help to engineer substances not only being multifunctional but also suitable interfaces between tissues. However, the utilization of pure conductive polymer-based hydrogel electronics is severely hindered by several issues such as rigid stiffness, limited injectability, and poor conductivity^12,13^. Although introducing metal nanoparticles (e.g., gold and silver nanoparticles) and carbon-based materials (e.g., carbon nanotubes and graphene) into hydrogels has been considered to be an effective technique to alter material conductivity for numerous applications^14–17^. Some characteristics, such as limited dispersibility and connectivity in an aqueous environment and structural rigidity, may limit their seamless interaction with biological tissues. Recently, there has been growing interest in liquid metals (LM), such as gallium–indium eutectic alloys (EGaIn: 75% Ga and 25% In w/w, with melting points at 15.7 °C). Considering the fluidic nature, high conductivity, chemical stability, biocompatibility, low viscosity, injectability, and self-healing property^18–20^, LMs can serve as flexible fillers in hydrogel networks. In contrast to the rigid metal nanoparticles, LMs provide unique features to create soft, injectable, and cytocompatible conductive materials for many biomedical applications, such as drug delivery and wearable electronics^19,21–23^.

For many applications, such as wearable bioelectronics^24^, wound dressing^25^, tissue engineering^26^, and drug delivery^27^, a hydrogel needs to adhere firmly to substrate surfaces under either dry or wet conditions. Many bioadhesive materials are complex mucilaginous mixtures containing proteins, sugars, and hydrophobic molecules^28–30^. Various types of chemical bonds and interactions resulted from the complex compositions can contribute to both the adhesion to a substrate and the cohesion in the network required for adhesive strength. Catechol-based adhesion of some marine organisms to almost any kind of surface under wet conditions has provided a valuable biomimetic source to develop bio-inspired adhesives^31–34^. However, several problems, such as sophisticated administration, slow curing^35^, the need for oxidant additive^36^, and low adhesion strength may hamper the actual applications of these biomimetic materials in clinically relevant scenarios.

We hypothesize that the diversity of reversible chemical bonds in a cross-linked network is important for a hydrogel material to possess substantial adhesive property, because energy dissipation during the rupture of adhesive bonds can be associated with many different interactions and processes^37^. We have recently reported an electroconductive hydrogel possessing a hybrid network composed of noncovalent and reversible covalent bonds^38^. The synergy among different types of chemical bonds, including covalent imine bond (Schiff base), hydrogen bond, π–π/anion–π/cation–π interaction, and electrostatic interaction, has resulted in reliable adhesive strength comparable to clinically used fibrin glue. The reversibility of these interactions has also led to self-assembled biomatrices, which can be used to encapsulate cells for 3D cell culture, as well as injectable materials for biocompatibility tests in mice.

We speculate that a higher adhesive strength could be achieved if the complexity of reversible bonds in the network can be further increased. Metal-polyphenol coordination^39^ and hydrophobic interactions^30^ have been found to play important roles in many bioadhesives. Introducing metal components into a conductive polymer matrix will also cause metal–π interactions, which will in turn influence the spatial arrangement and assembly at the nano and molecular scales, thus, affecting the electric properties, such as electroconductivity, resistance, and capacitance^40,41^. To construct such a complex system is very attractive for understanding the underlying mechanisms of bioadhesives through a bottom-up approach as well as for the development of smart artificial materials. However, efficacy and reliability in synthesis represent the major challenge. With the conventional linear strategy to synthesize sophisticated structures, overall yield quickly drops with each reaction step. A representative example is dendrimer synthesis, even with very simple and efficient chemical reactions, growth of dendrimer to higher generations will inevitably lead to incomplete conversion and cause defects^42^. Therefore, a convergent strategy, which is highly favorable for the total organic synthesis, will be even more attractive for macromolecule synthesis because of the difficulty to separate, purify, and characterize the polymer intermediates and final products.

In this work, we aim to develop a self-assembled hydrogel by facile mixing of polysaccharides, conductive biopolymers, and liquid metal nanodroplets. By implementing the concept of convergent synthesis, a highly complex reversible polymer network can be synthesized, including a reversible covalent bond and a large variety of noncovalent interactions. All precursors can be individually synthesized in one to two reaction steps, and assembled to form hydrogels in a stepwise fashion. It is important to note that the structure and concentration of each intermediate can be varied, leading to a modular system with tunable mechanophysical and biochemical properties. The bio-inspired elaborate network exhibits multifunctionality, including enhanced adhesiveness and electroconductivity, injectability, and compatibility with 3D printing, 3D cell culture, and in vivo magnetic resonance imaging (MRI) and computed tomography (CT) imaging. The high biocompatibility of the materials is demonstrated in immunocompetent mice.

## Results

**Fig. 1:**
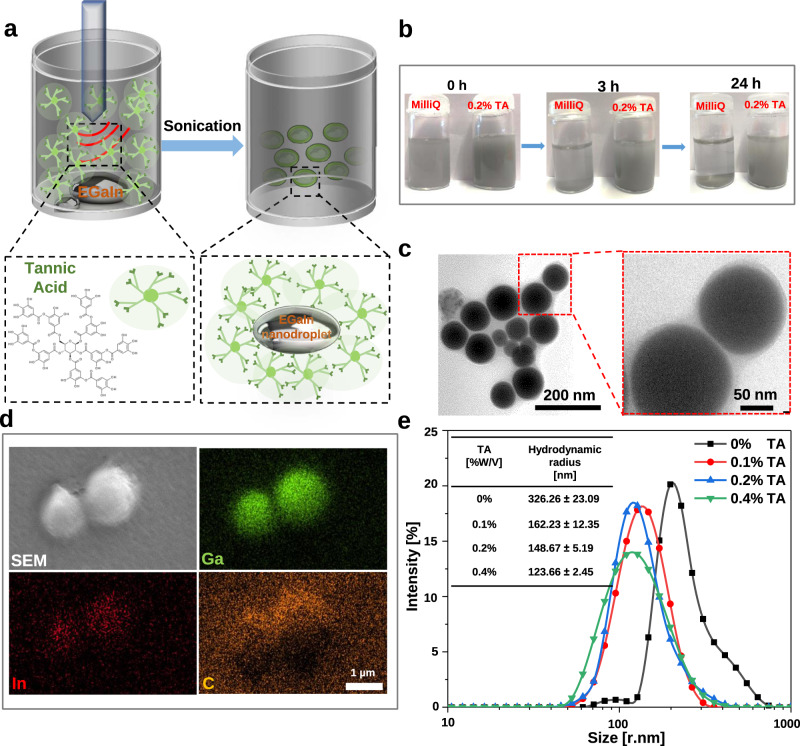
Production of EGaIn nanodroplets. **a** Schematic illustration of sonicating eutectic gallium–indium (EGaIn) in tannic acid (TA) solution and EGaIn droplets shelled by tannic acid. **b** Photos for time-dependent dispersion of liquid metal (LM) in water and tannic acid solution after ultrasonication. **c** TEM images of tannic acid-coated liquid metal nanodroplets (TA-LM) with core–shell nanostructure. A representative image of three individual experiments is shown. **d** Cryo-scanning electron microscopy (Cryo-SEM) image of LM droplets. EDS mapping of Ga (gallium), In (indium), and C (carbon). A representative image of three independent samples is shown. **e** DLS analysis of LM droplets. Inset table: feeding compositions of LM/TA mixture solution and LM droplets size.

Eutectic gallium–indium (EGaIn) (75.5% gallium and 24.5% indium) was used to generate LM nanodroplets by ultrasonication treatment. However, the EGaIn nanodroplets are not stable and can oxidize and de-alloy under aqueous conditions^22^. To overcome this problem, we used a facile method to prepare LM nanodroplets by sonication in the presence of tannic acid (TA) (Fig. 1a). TA is a corrosion inhibitor through interaction with metals and alloys^43^. An opaque slurry was generated after 30 min of sonication, and stable EGaIn nanodroplets were obtained after washing and size-grading. The transmission electron microscopy (TEM) images demonstrate that the LM nanodroplets were well-dispersed (Fig. 1c) and showed a core–shell structure with TA as hydrophilic coating. In the absence of TA, there is no core–shell structure that can be observed via TEM (Supplementary Fig. 1) and the LM nanodroplets in solution were not stable and would aggregate and sink to the bottom after 1 h (Fig. 1b). The element distribution mapped by energy-dispersive X-ray spectroscopy (EDS) confirmed the composition of LM nanodroplets, demonstrating the presence of gallium (Ga), indium (In), and carbon (C) (Fig. 1d). The TA coating was also confirmed via Fourier transform infrared spectroscopy (FTIR), and the characteristic vibration bands of the phenol group at 1202 cm^−1^ (C–O) and the aromatic group at 1613 cm^−1^ (C=C) in LM-TA nanodroplet have emerged (Supplementary Fig. 2). The size of LM nanodroplets can be adjusted by TA concentration and assessed by dynamic light scattering (DLS). The more TA was added, the smaller the size of LM droplets could be obtained after 30 min of ultrasonication (Fig. 1e). Moreover, aggregation was less likely to occur at low TA-to-LM ratio. In addition to a corrosion inhibitor, TA can serve as a stabilizing reagent to prevent the LM nanodroplets from aggregation during sonication.

### Convergent synthesis of the polymers and hydrogel fabrications

**Fig. 2:**
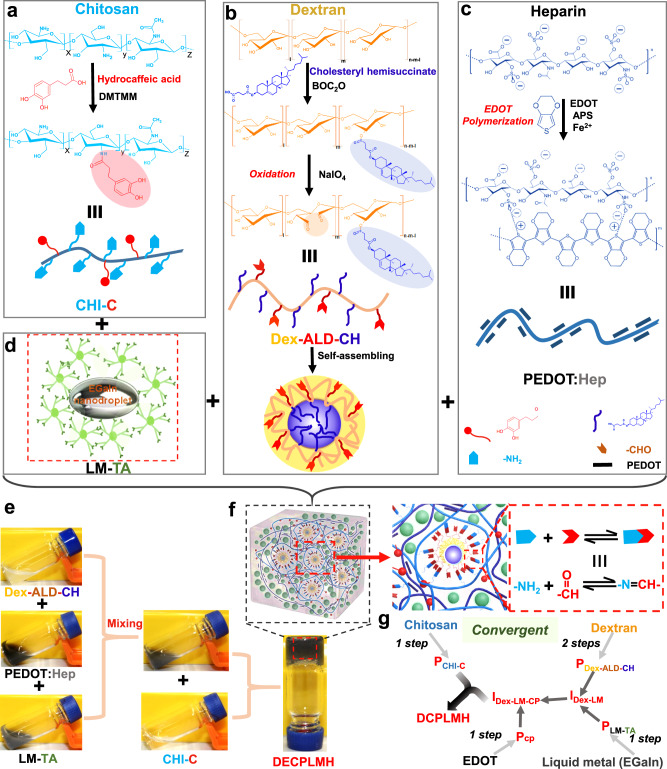
Characteristics of the dual cross-linked electroconductive hydrogels. **a** Schematic illustration of synthesis of catechol-conjugated chitosan (CHI-C). **b** Schematic illustration of synthesis of cholesteryl hemisuccinate (CH) conjugated oxidized dextran (Dex-ALD-CH). **c** Schematic illustration of synthesis of poly(3,4-ethylenedioxythiophene)-heparin (PEDOT:Hep). **d** Liquid metal-tannic acid (LM-TA) nanodroplets. **e** Representative hydrogel formation by homogenous mixing of Dex-ALD-CH+ PEDOT:Hep+LM-TA with CHI-C solutions in glass vial. **f** Schematic illustration of the cross-linked polymer networks formed by dynamic Schiff-base bond in dynamic electroconductive biopolymer/liquid metal hydrogels (DECPLMH). **g** Schematic illustration of convergent synthesis methodology to form the DECPLMH.

We designed and synthesized dynamic electroconductive biopolymer/liquid metal hybrid hydrogels (DECPLMH), which were formed by mixing TA-coated LM nanodroplets (P_LM-TA_) with catechol-functionalized chitosan (P_CHI-C_), cholesteryl and aldehyde-modified dextran (P_Dex-ALD-CH_), and PEDOT:Hep (P_cp_) (Fig. 2a–e). Instead of a linear synthesis strategy that will generate precursor P_n_ after *n* steps of reaction followed by cross-linking to produce a complex hydrogel network (Supplementary Fig. 3), we utilized the convergent strategy to synthesize the DECPLMH (Fig. 2g). Following the linear strategy, it is practically difficult to purify and characterize macromolecules after many reaction steps. Moreover, the presence of a large variety of interacting chemical groups would not only require a protection/deprotection strategy (thus more reaction steps) but also cause unforeseeable solubility/aggregation problems. While a convergent synthesis is highly favorable, it would be even more attractive if the later steps contain no chemical reaction. Thus, all precursors can be produced by simple chemical reaction(s) and analyzed/controlled/defined. Furthermore, if the final gelation can be achieved through noncovalent self-assembly, cell-laden matrices can be produced in a chemical reaction-free manner.

To produce the precursors P_CHI-C_, P_Dex-ALD-CH_, and P_CP_, we have chosen chitosan, dextran, and heparin as starting compounds, respectively. The polysaccharides can provide not only basic biocompatible structures but also a large number of hydroxyl groups as H-bond donors and acceptors present in many bioadhesives^44^. P_CHI-C_ was synthesized via coupling hydrocaffeic acid to the primary amino groups in chitosan using 4-(4,6-Dimethoxy-1,3,5-triazin-2-yl)-4-methylmorpholinium chloride (DMTMM) (Fig. 2a). The FTIR spectrum confirmed the appearance of the characteristic peaks for the functional groups of amide C=O (∼1646 cm^−1^), aromatic C=C (∼1517 cm^−1^), and phenol OH (∼1309 cm^−1^), demonstrating the successful conjugation of hydrocaffeic acid to chitosan (Supplementary Fig. 4).

NMR measurements showed a catechol substitution degree of ∼9% for P_CHI-C_ (Supplementary Fig. 5). Lipophilic-precursor P_Dex-ALD-CH_ was synthesized by conjugating cholesteryl hemisuccinate to dextran, followed by oxidation to generate aldehyde groups (Fig. 2b). FTIR measurement showed a newly formed peak at 1733 cm^−1^, corresponding to the stretching vibration of the aldehyde group, C=O bond in P_Dex-ALD-CH_ (Supplementary Fig. 6). NMR measurements indicate a cholesteryl substitution degree of ∼10% for Dex-CH (Supplementary Fig. 7). Synthesis of the electroconductive biopolymer precursor P_CP_ (PEDOT:Hep) was carried out by oxidative polymerization of EDOT with highly sulfated polysaccharide heparin as dopant^38^ (Fig. 2c) and validated via NMR (Supplementary Fig. 8). As described before, P_LM-TA_ was produced by sonicating EGaIn in 0.2% (%W/V) TA ethanol solution (Fig. 2d). Overall, while P_CHI-C_, P_LM-TA_, and P_CP_ were synthesized in one step, P_Dex-ALD-CH_ was synthesized in two steps.

**Fig. 3:**
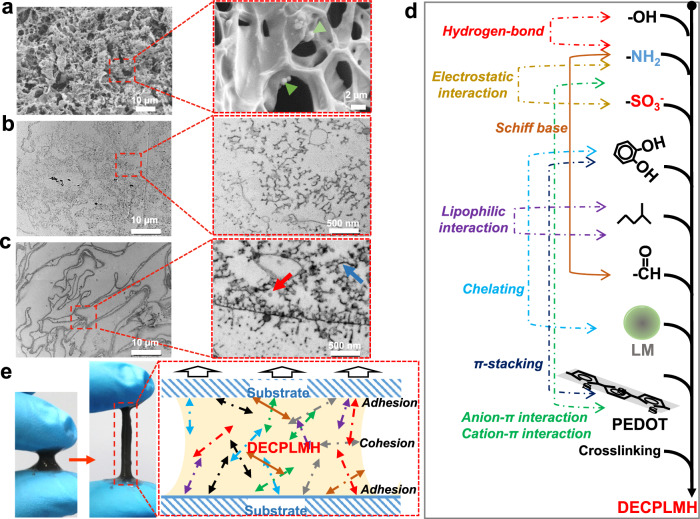
Illustration of multiple types of reversible chemical bonds that could be important for the development of potent bioadhesives. **a** Cryo-SEM images of DECPLMH networks. The arrows in samples indicate LM nanodroplets in the DECPLMH networks. A representative image of three samples is shown. **b** Transmission electron microscope (TEM) image of DECPH networks. A representative image of three individual experiments is shown. **c** TEM image of DECPLMH networks. The arrows in samples indicate LM nanodroplets in the DECPLMH networks. A representative image of three individual experiments is shown. **d** Schematic illustration of multiple types of interactions, which contribute to form the dynamic electroconductive biopolymer/liquid metal hydrogels (DECPLMH). **e** Photos of DECPLMH are sketched with two fingers and schematic illustration of multiple types of interactions, which contribute to the cohesion and adhesion property of the DECPLMH.

As shown in Fig. 2e, DECPLMH can be formed by stepwise mixing the four polymer precursors. FTIR spectrum demonstrated a peak at 1644–1656 cm^−1^, corresponding to the Schiff base (-C=N-), which is formed between the aldehyde groups of P_Dex-ALD-CH_ and the amino groups of P_CHI-C_ (Fig. 2f and Supplementary Fig. 9). The S=O (∼1150 cm^−1^) and C–O–C (∼1130 cm^−1^) bands demonstrate the incorporation of P_CP_ into the hybrid matrix (Supplementary Fig. 9). The convergent and modular system can also be used to produce materials with reduced complexity of cross-linking chemistry (Fig. 2g). For example, by mixing P_CHI-C_ (2%, %W/V) and P_Dex-ALD-CH_ (2%, %W/V), the Schiff-base cross-linked dynamic hydrogel (DH) can be synthesized. By mixing P_CHI-C_ (2%, %W/V) with premixed P_CP_ (0.8%, %W/V) and P_Dex-ALD-CH_ (4%, %W/V), dynamic electroconductive hydrogel (DECPH) can be formed. P_CHI-C_ (2%, %W/V) can also assemble with I_Dex-LM_ (premixed 2% P_LM-TA_ and 4% P_Dex-ALD-CH_, %W/V), leading to dynamic LM hydrogel (DLMH). The Cryo-scanning electron microscope (Cryo-SEM) and transmission electron microscope (TEM) images illustrated that DECPLMH possessed a highly porous structure, with a homogeneous distribution of spherical LM nanodroplets (Fig. 3a–c). In the matrix network of DECPLMH (Fig. 3d), many different reversible bonds, such as covalent imine bond (Schiff base) and noncovalent interactions (electrostatic interaction, hydrogen bond, hydrophobic interaction, π–π stacking, anion/cation–π interaction, complexation between liquid metal and polyphenol group) are involved in cross-linking to form the dynamic hydrogel networks. Degradation of the materials can be mediated by Schiff-base hydrolysis and dissociation of the non-covalently cross-linked building blocks from the reversible network. To evaluate the stability of the resulting diversified reversible network, we performed in vitro degradation studies of DH and DECPLMH in PBS. The in vitro degradation tests were carried out in PBS at 37 °C with or without 10 unit ml^−1^ lysozyme. As shown in Supplementary Fig. 11, in the absence of hydrolytic activity of enzymes, both DH and DECPLMH showed very slow degradation in PBS (more than 70% mass of DECPLMH left after 21 days of incubation), while the degradations can be remarkably accelerated by lysozyme treatment. We hypothesized that the diversity in chemical functionality and the resulting network complexity can contribute to the injectability, the adhesion of the material to the substrate surfaces, and the cohesion within the network. Both adhesion and cohesion are essential for an adhesive material (Fig. 3e), which we will demonstrate in the following results.

### Rheological characterization of hydrogels

**Fig. 4:**
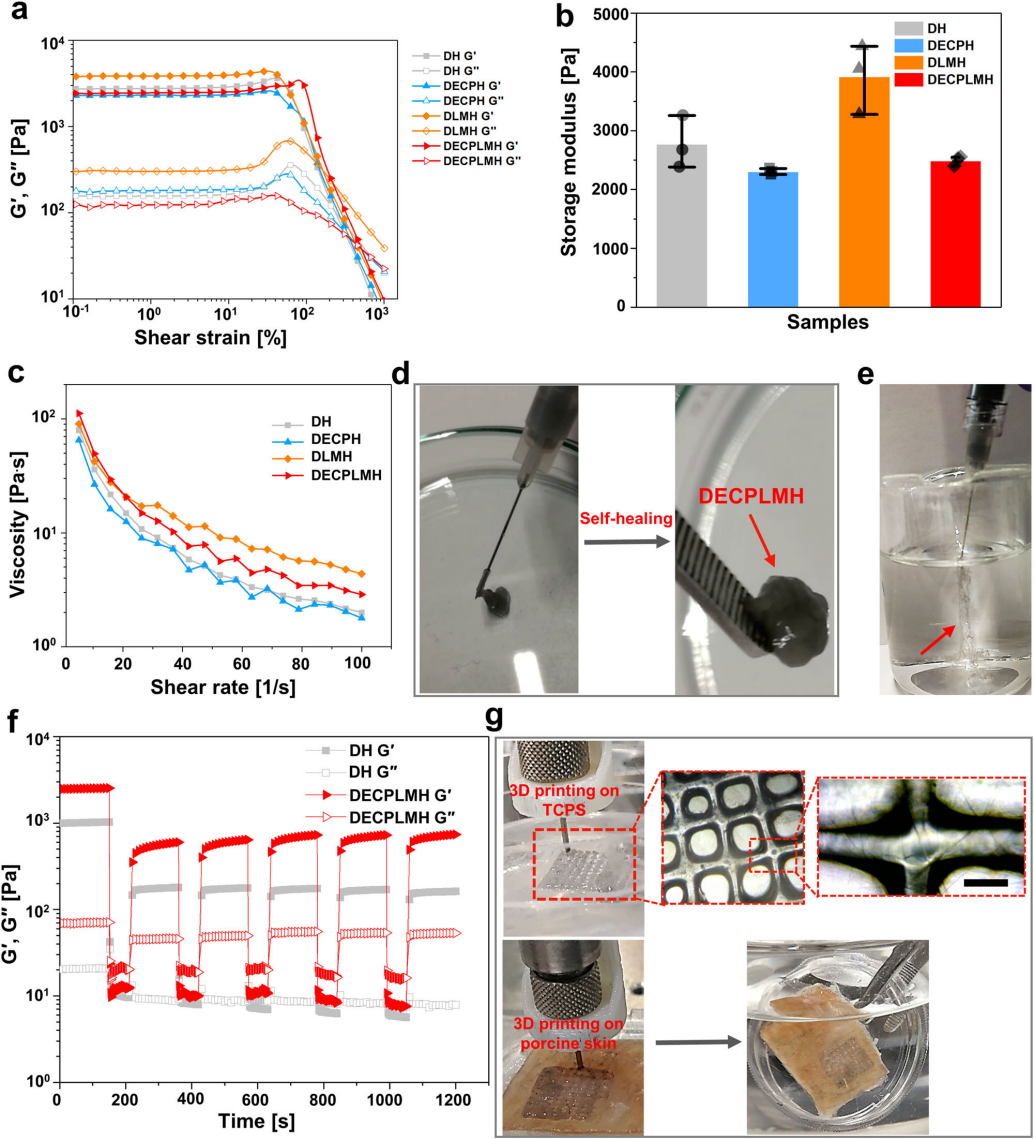
Rheological properties of the electroconductive hydrogels. **a** Amplitude sweep performed with the shear strain increasing from 0.1% to 1000%. **b** The storage modulus of hydrogels at 10% shear strain (*n* = 3 hydrogel samples, mean ± SD). **c** Continuous flow experiments showing the viscosity of the hydrogels plotted against the shear rate. **d** Injection of a hydrogel via a syringe with 27G needle onto a plastic plate. The injected dynamic electroconductive biopolymer/liquid metal hydrogels (DECPLMH) maintain their structural integrity when gripped and sticks to the tweezer (indicated by the arrows). **e** The DECPLMH was syringe-injectable and thin filaments (indicated by the arrows) were extruded into PBS solution. **f** Self-healing property of the hydrogels when the alternate step strain was switched from 1 to 1000%, indicated by the recoverage of the elastic modulus. **g** 3D printing of a DECPLMH, which can adhere to the TCPS (tissue culture plastic surface) (upper panel), and the magnification of the printed fiber overlapping area. Scale bar = 200 µm. A representative image of three individual samples is shown. 3D printing in situ on porcine skin (the bottom panel), and remaining stable when holding the dish vertically in the 0.01 M PBS solution.

Strain-dependent oscillatory rheology of four dynamic hydrogels with reversibly cross-linked networks of different complexity displayed broad linear viscoelastic regions in addition to network failure at very high strains (>10% shear strain), indicating a broad processing regime and shear-thinning behavior (Fig. 4a, b). The non-Newtonian behavior of shear-thinning was further demonstrated using steady shear measurements, showing a gradual decrease of viscosity upon increasing shear rate (Fig. 4c). While the shear-thinning property is required for an injectable polymer material to be extruded through high gauge needles, self-healing reflects the ability of the network to reform after extrusion^45^. It is important to note that the rheological property of self-healing is different from the regeneration property of biological systems. To develop biomaterials with shear-thinning and self-healing features is important for their applications through injection, a minimally invasive process for many biomedical utilities^46–48^. Step-strain measurements were performed to test the self-healing property of hydrogels. At a high strain of 1500%, all hydrogels yielded, while at a low strain of 1%, the hydrogels rapidly recovered to their hydrogel state (Fig. 4f). This behavior was stable over many cycles, demonstrating the robust self-healing ability.

DECPLMH (1% P_CHI-C_ + 0.2% P_CP_ + 1% I_Dex-LM_, W/V) can be easily extruded through a 27-gauge needle (inner diameter = 0.21 mm) onto a glass dish and reform bulk gel again within 5 min (Fig. 4d, Supplementary Movies 1 and 2). The hydrogel can quickly reform once exiting the needle not only on the dry surface but also in aqueous solution, thus suitable for underwater injection (Fig. 4e, Supplementary Movie 3), which is a desirable property for surgical practice. We then tested whether the injectable materials can also be used for microextrusion 3D-bio-printing. As shown in Fig. 4g, the DECPLMH (1% P_CHI-C_ + 0.2% P_CP_ + 1% I_Dex-LM_, %W/V) can be printed as three layers of grid structure on a plastic dish surface (Supplementary Movie 4) as well as on a wet porcine skin surface (Movie S5). The printed materials bound tightly to the substrates even when immersed in 0.01 M PBS (Supplementary Movies 6 and 7). This observation suggested that the hybrid hydrogel fulfills the requirements not only for underwater injection but also to be adhesive for biological tissue-relevant wet surfaces.

### Adhesive properties of the hydrogels

**Fig. 5:**
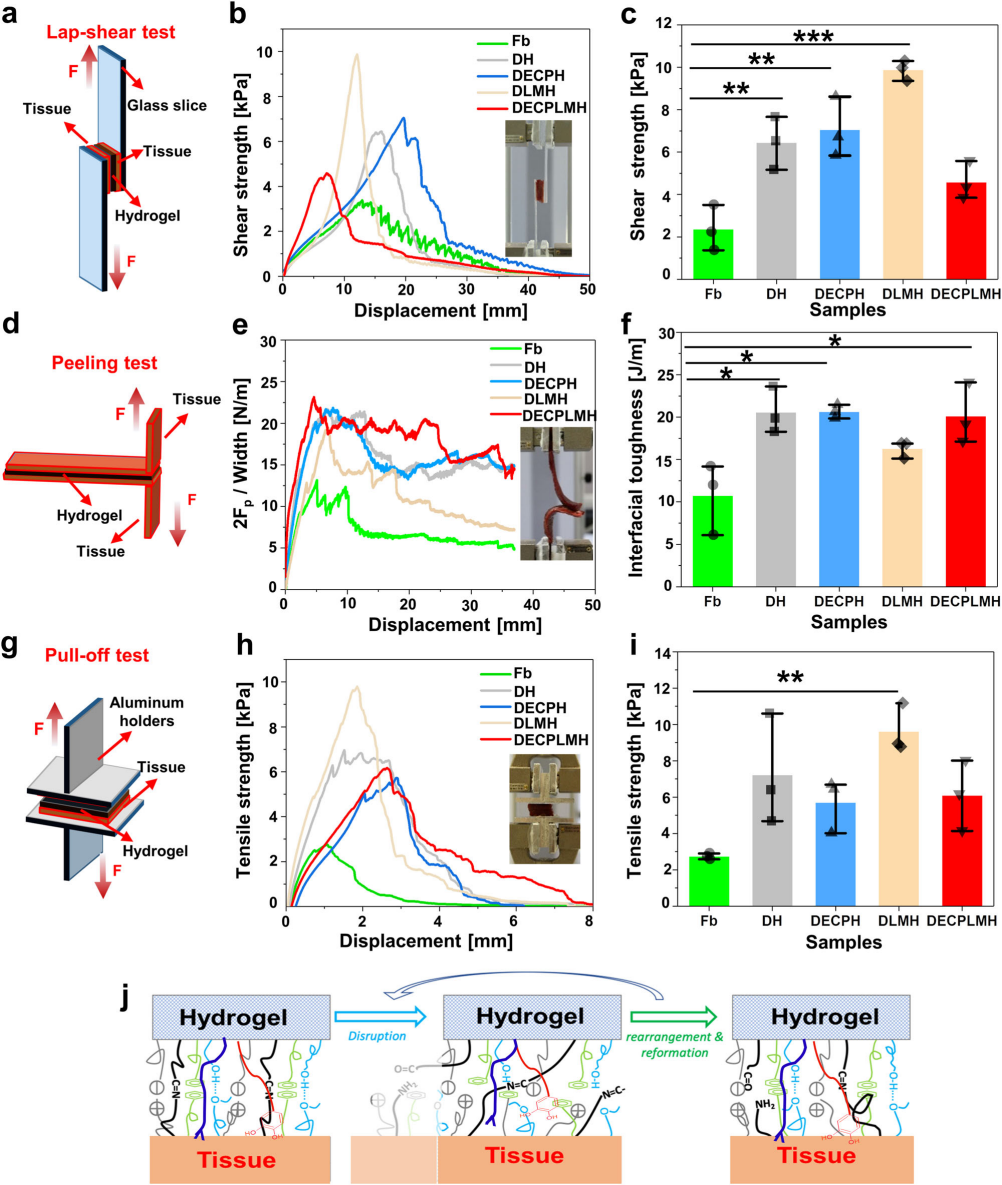
The adhesive property of electroconductive hydrogels. **a** Setup for measurement of shear strength. **b** Adhesion lap-shear testing of hydrogels, where the shear strength is plotted against the displacement. Inset: photo of the experimental setup. **c** The shear strength of the hydrogels (*n* = 3 independent hydrogel samples, mean ± SD, one-way ANOVA, Tukey multiple pairwise comparison test for multiple comparisons to Fb gel, DH vs Fb **p = 0.00628, DECPH vs Fb **p = 0.00236, DLMH vs Fb ***p=0.0000495). **d** Setup for measurement of peeling test. **e** Curve of double peeling force per adhesive width between the hydrogels and porcine myocardium tissues (inset: photos of the peeling process). **f** The calculated interfacial toughness between the hydrogels and porcine myocardium tissues (*n* = 3 independent hydrogel samples, mean ± SD, one-way ANOVA, Tukey multiple pairwise comparison test for multiple comparisons to Fb gel, DH vs Fb *p = 0.0109, DECPH vs Fb **p = 0.0105, DECPLMH vs Fb *p = 0.0711). **g** Setup for measurement of tensile strength. **h** Adhesion pull-off testing of hydrogels, where the adhesive strength is plotted against the extension (~distance). Inset: Detail of the experimental setup. **i** The pull-off strength of the hydrogels (*n* = 3 independent hydrogel samples, mean ± SD, one-way ANOVA, Tukey multiple pairwise comparison test for multiple comparisons to Fb gel, **p = 0.01). **j** Illustration of multiple types of reversible chemical bonds formed between the DECPLMH and tissue interface. Data are displayed as mean ± SD (*p < 0.05, **p < 0.01, ***p < 0.001).

We evaluated the adhesion of the hydrogels by carrying out three different types of force experiments to measure (1) the shear strength by lap-shear tests (Fig. 5a–c), (2) interfacial toughness by peel tests (Fig. 5d–f), and (3) tensile strength by pull-off tests (Fig. 5g–i). We used pre-wetted porcine myocardium as a model substrate, owing to its mechanical robustness, resemblance to human muscle tissue, and relevance to the clinical use under wet conditions. Clinically applied fibrin glue was used as a positive control compared to hydrogels.

The lap-shear tests measured the ability of an adhesive to withstand stresses and separation forces set in a plane. The shear forces are exerted while moving two substrates parallely toward opposite directions (Fig. 5a). The stress values were calculated by dividing the maximum shear force by the corresponding overlapping area of two substrates joined by adhesive material (Fig. 5b). We have previously shown that a hybrid hydrogel network mediated by H-bond and Schiff base could exhibit adhesive strength corresponding to about 50% of that of fibrin glue^38^. With the incorporation of polyphenol groups (causing π–π interaction) and cholesteryl groups (causing lipophilic interaction), the resulting DECPH hydrogel exhibited a 3-time higher shear strength compared to fibrin glue. Remarkably, by including LM nanodroplets into the network (causing complexation between metal and polyphenol groups), a further increase of shear forces was measured for DLMH hydrogel, exhibiting a shear strength of 10 kPa. Increasing the network heterogeneity can cause a moderate opposite effect, as shown in the lap-shear measurements with DECPLMH hydrogel.

In addition, peeling (Fig. 5d–f) and pull-off (Fig. 5g–i) tests with fibrin glue and hydrogels on porcine myocardium tissues were conducted. In these measurements, all hydrogels adhered stronger compared to fibrin glue. A high percentage of P_LM-TA_ could interact with the amino groups of P_CHI-C_ with hydrogen bonding, thus inhibiting the formation of neutral imine bond with the aldehyde groups of P_Dex-ALD-CH_ and shifting the hybrid system from covalent to noncovalent cross-linking caused weakened cohesion of the hydrogel network, as reflected by the reduced adhesive strength (Supplementary Fig. 13). During the rupture of a reversible network, energy could be dissipated more efficiently through breaking bonds of different strength^37^, while successive disruption, rearrangement, and reformation of these interactions resulted in the adhesive property (Fig. 5j). It is important to note that the hybrid networks are modular systems, and the structure and concentration of each component can be varied. To orchestrate the contributions of different reversible bonds by tuning these components could lead to further optimization of the adhesive material.

### Electrical properties of the hydrogels

**Fig. 6:**
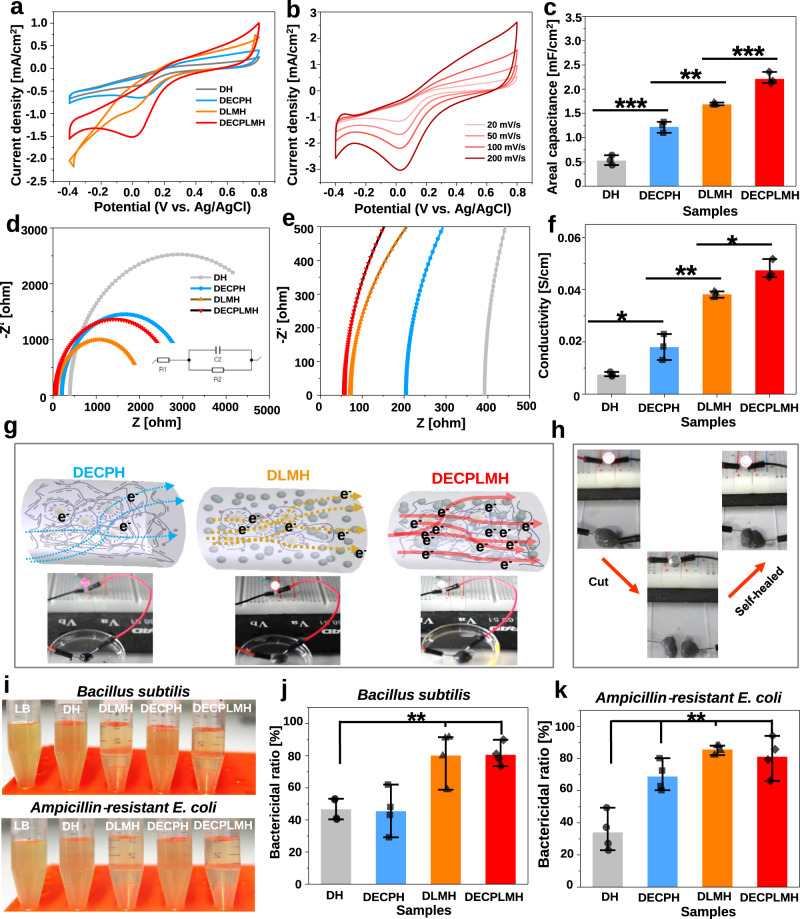
Electroconductive and antibacterial properties of the electroconductive hydrogels. **a** Cyclic voltammograms (current density vs the potential) of hydrogels in PBS at a scan rate of 50 mV·s^−1^. **b** Cyclic voltammograms (current density vs the potential) of hydrogels in PBS at different scanning speed. **c** Areal capacitance of different hydrogels (*n* = 3 independent hydrogel samples, mean ± SD. One-way ANOVA, Tukey multiple pairwise comparison test for multiple comparisons, DH vs DECPH ***p = 0.000125, DECPH vs DLMH **p = 0.00199, DLMH vs DECPLMH ***p = 0.000891). **d** Electrochemical impedance spectroscopy of hydrogels (inset: equivalent circuit). **e** Nyquist plot of the hydrogels. **f** The conductivity of hydrogels (*n* = 3 independent hydrogel samples, mean ± SD. One-way ANOVA, Tukey multiple pairwise comparison test for multiple comparisons, DH vs DECPH *p = 0.0158, DECPH vs DLMH **p = 0.00254, DLMH vs DECPLMH *p = 0.0323). **g** Schematic of hydrogel structures, including dynamic electroconductive polymer hydrogel (DECPH), dynamic liquid metal hydrogel (DLMH), dynamic electroconductive biopolymer/liquid metal hydrogels (DECPLMH). The arrows describe the proposed electron (e^−^) transfer passing through each hydrogel, including a solid line for continuous flow and dashed line for discontinuous flow. LED emitting tests in electrical circuit serially connected with the various hydrogels. **h** Self-healing properties of the hydrogel: photo demonstrating the good conductivity of the hydrogel after self-healing which based on the LED intensity. **i** Photos of *Bacillus subtilis* and *Ampicillin‐resistant E. coli* solutions co-cultured with the hydrogels after 1 day. **j** The bactericidal ratio of the hydrogels to *Bacillus subtilis* (*n* = 4 biologically independent samples. One-way ANOVA, Tukey multiple pairwise comparison test for multiple comparisons to DH gel, DLMH **p = 0.00726, DECPLMH **p = 0.00459). **k** The bactericidal ratio of the hydrogels to Ampicillin‐resistant *E. coli* (*n* = 4 biologically independent samples. mean ± SD. One-way ANOVA, Tukey multiple pairwise comparison test for multiple comparisons to DH gel, DECPH **p = 0.00346, DLMH **p = 0.00143, DECPLMH **p = 0.00131). Data are displayed as mean ± SD (*p < 0.05, **p < 0.01, ***p < 0.001).

The electrochemical properties of conductive hydrogels were studied using a three-electrode system (Supplementary Fig. 17a). The cyclic voltammetry (CV) curves of DECPH and DECPLMH hydrogels exhibit reduction peaks between 0.0–0.1 V, typical for PEDOT-based materials (Fig. 6a). The oxidation peak of PEDOT (between 0.5 and 0.7 V) could not be detected, presumably because of the presence of polyphenol groups. In the absence of PEDOT:Hep (P_CP_), the reduction peak between 0.0–0.1 V was diminished for DH and DLMH. Figure 6b shows the rate-dependent CVs of DECPLMH with the potential window of −0.2 to 0.8 V vs. Ag/AgCl reference electrode at scan rates of 20, 50, 100, and 200 mV s^−1^ (Fig. 6b). The remarkable difference in CV curves among different materials also indicates their difference in capacitance. The areal capacitances of materials have been calculated from the CV curves (Fig. 6c), and DECPLMH has shown the highest capacitance. Electrochemical impedance spectroscopy (EIS) was used to further study the electrical performance of the materials (Supplementary Fig. 17b). As shown in Fig. 6d, Electrochemical impedance spectroscopy (EIS) is presented as Nyquist plots, and all materials exhibit semicircles typical for the equivalent electrical circuit of Randles cell. The equivalent series resistance (ESR) of DECLMH hydrogel extracted from high frequency (100 kHz) is estimated to be 62 *Ω*, which is much lower than that of the non-electroconductive DH hydrogel (Fig. 6e). The conductivity of DECPLMH hydrogel was higher than that of the DECPH hydrogel and DLMH when the same corresponding concentrations of P_CP_ or P_LM_ were used (Fig. 6f). The electroconductivity measurements were further performed with varied concentrations of LM in DECPLMH. As shown in Supplementary Fig. 15, the corresponding increase of conductivity can be achieved when increasing LM from 0.1 to 5% (W/V). However, the improvement is not very drastic, showing only two times enhanced conductivity upon increasing LM from 0.2 to 5%. Moreover, the DECPLMH with 5% (W/V) LM is very brittle, thus not suitable for gel injection via 27G needle and adhesive applications.

In order to understand the difference in electrical properties between different materials, we have imaged the hydrogels with Cryo-SEM and TEM. Whereas the inter-connected porous structures at the µm scale were similar to each other, as revealed by Cryo-SEM (Supplementary Fig. 10), large differences at the nm scale between the materials were observed in the TEM images. The sulfated conductive polymer could be imaged after uranyl acetate staining, and LM nanodroplets could be directly visualized (Fig. 3c and Supplementary Fig. 12b). While the conductive polymer formed a loosely connected network in DECPH (Fig. 3b), the LM nanodroplets in DECPLMH were situated at the outer shells (Fig. 3c). Remarkably, the combination of two different types of conductive materials in DECPLMH resulted in dense shell structures assembled through the interaction between PEDOT:Hep and LM nanodroplets. The cross-linked LM nanodroplets and PEDOT:Hep in the hydrogel network of DECPLMH enabled continuous electrical flow (solid red line, Fig. 6g), as well as enhanced electrical energy storage, as reflected by the measured impedance and areal capacitance. The difference in electroconductivity between different materials could also be tested by inserting the hydrogels as resistors into a direct current (DC) circuit (Fig. 6g). By applying a voltage (3 V), significant difference in LED brightness was observed: DECPLMH > DLMH > DECPH. The conductivity of hydrogels in DC was characterized by *I*–*V* measurement (−3.5 to 3.5 V) via electrochemistry station with linear sweep voltammetry mode (scanning speed: 200 mv/s) (Supplementary Fig. 28), which demonstrated again that the LM conductive hydrogels are not simple resistors but contain both resistance and capacitance properties, as shown by the linear and nonlinear regions in I–V curves. The DECPLMH exhibits the highest current at 3.0 V, in good agreement with the light intensity difference. Moreover, as the hydrogels possess both self-healing and adhesive properties, the brightness of LED in DC circuit could be fully recovered after putting two divided pieces together (Fig. 6h). As shown in Supplementary Fig. 29a, the current response of DECPLMH during two cycles of cutting and self-healing was followed. The current decreased to zero after cutting and recurred after removing the coverslip because of the hydrogel’s self-healing capability. To further illustrate the self-healing process, bright filed images of the self-healing process of DECLMH hydrogel are shown in Supplementary Fig. 29b.

### Anti-microbial activity and biocompatibility of the hydrogels in vitro

As microorganisms and mammalian cells can respond differently to various chemical reagents, many substances have proven to be anti-microbial without cytotoxic effects to mammalian cells.

Several biocompatible components used in the hydrogel adhesives, including chitosan, tannic acid, and LM, have anti-microbial activity^43,49,50^. We investigated the growth of *Bacillus subtilis* (Gram-positive bacteria) and ampicillin-resistant *Escherichia coli* (Gram-negative bacteria) in the presence of various hydrogel adhesives. Figure 6i shows bacteria suspensions cultured with the hydrogels for 24 h. The suspensions of negative control, DH, and DECPH hydrogels were turbid, whereas those of DLMH and DECPLMH hydrogels were clear. The optical density (OD 600 nm) values were measured. The presence of LM nanodroplets (in DLMH and DECPLMH hydrogels) resulted in high anti-microbial potency as compared to the DH and DECPH hydrogels (Fig. 6j and k). The bactericidal ratios of DECPLMH for *B. subtilis* and *E. coli* were 82% and 79%, respectively. The hydrogels could effectively inhibit both G− and G+ bacteria. Because the materials caused neither toxic effect to mammalian cells nor adverse reaction upon injection in immunocompetent mice (as shown in the following sections), the anti-microbial activity is highly desirable for their application for cell culture and clinical utilities.

**Fig. 7:**
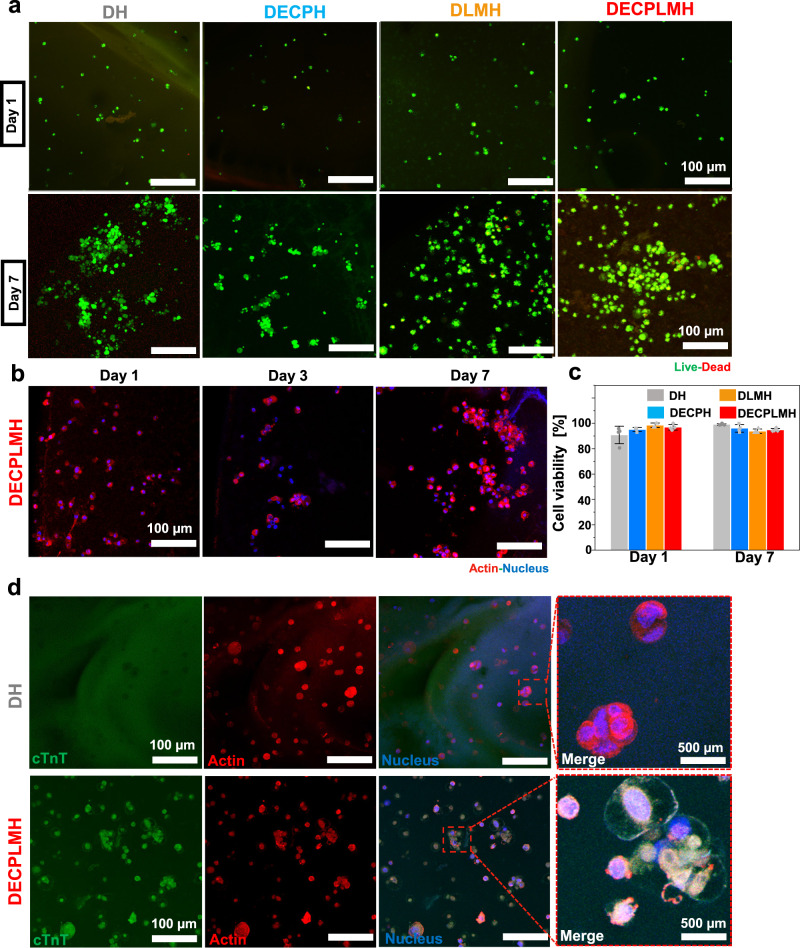
Biocompatibility of the electroconductive hydrogels in vitro. **a** Live-dead staining of mouse myoblasts cultured in hydrogels at day 1 and day 7, with calcein-AM (green; viable) and ethidium homodimer-1 (red; dead). Scale bar: 100 μm. A representative image of three biological replicates is shown. **b** Immunofluorescence staining of the cells cultured in DECPLMH at day 1, day 3, and day 7 of the cell nucleus (blue; Hoechst) and actin (red; phalloidin). Scale bar: 100 μm. A representative image of three biological replicates is shown. **c** The cell viability of the C2C12 cells after 3D culture at day 1 and day 7 (*n* = 3 biologically independent samples, mean ± SD). **d** cTnT (green) and actin (red) immunofluorescence staining of C2C12 cells cultured in DH and DECPLMH hydrogels with differentiation medium for 10 days. A representative image of three biological replicates is shown.

We investigated the culture of myoblast cells encapsulated in the adhesives. Mouse skeletal myoblast C2C12 cells were embedded in DH, DECPH, DLMH, and DECPLMH hydrogels (Fig. 7a). The cells proliferated and formed spheroid structures after 7 days of culture (Fig. 7b). Cell viability was assessed by live/dead staining 1st and 7th day after cell encapsulation. All hydrogels exhibited high cytocompatibility showing >90% cell viability (Fig. 7c), while a few cell deaths could be observed in the LM nanodroplets-containing DLMH and DECPLMH hydrogels.

We further performed the human primary mesenchymal stromal cell (MSC) and mouse L929 fibroblast cell long-term cultures (21 days) both in 2D and 3D. As shown in Supplementary Fig. 18 of 3D cultures of encapsulated cells, the live-dead staining demonstrated that both MSCs and L929 cells exhibited high cell viability and continued to proliferate in both DH and DECPLMH. The nuclei and actin staining showed that both types of cells have grown into large clusters (Supplementary Fig. 19). The MSCs and L929 cells also exhibited high cell viability and continued to proliferate (Supplementary Fig. 22) over the 21-day of 2D cell-gel co-culture, when the cells were seeded on culture plastic in the presence of hydrogel adhesive (30 µl). After 7 days, the cells spread over the plastic areas and started to grow onto the hydrogel materials, causing the degradation of gels. After 3 weeks, both plastic and hydrogel surfaces were largely covered cells, especially for the fast proliferating L929 cells.

When proliferating to cover the surface, the growth rates decreased gradually. The EdU staining assays (Supplementary Figs. 20 and 21) demonstrated the reducing number of proliferating cells, in good agreement with the reducing rate of increasing cell number. No obvious difference has been observed between DECPLMH and DH (Supplementary Fig. 23). Overall, our results show that the DECPLMH hydrogel possesses good cytocompatibility when exposed to various types of cells (C2C12, MSCs, and L929) in long-term (21 days) 2D (cell-material contacts) and 3D cultures.

When subjected to differentiation condition, myogenesis of myoblasts into mature and multinucleated myotubes in the low and high electroconductive DH and DECPLMH hydrogels were evaluated by immunofluorescence staining for the myogenic differentiation marker troponin T (Fig. 7d). Remarkably enhanced troponin T expression was observed for cells in the DECPLMH hydrogel after 3 days, but not in the DH hydrogel. After 3D cell culture at both differentiation condition (10 days) and long-term (21 days) proliferation conditions, partial degradations (as PEDOT and LM are not biodegradable) of both materials were noticed because of the polymer hydrolysis by enzymes secreted by cells, in good agreement with the following in vivo imaging analysis. Consequently, the DECPLMH hydrogel became more darkish because of concentrating the non-degradable PEDOT in the remaining materials, interfering with the confocal fluorescence imaging. Nevertheless, enhanced expression of troponin T in both types of materials could be measured, while multinucleated myotubes were detected only in the DECPLMH hydrogel (Supplementary Fig. 23). The electroconductive adhesive hydrogel could accelerate myogenesis of myoblasts into mature and multinucleated myotubes.

### Biocompatibility and imaging of hydrogels in vivo

**Fig. 8:**
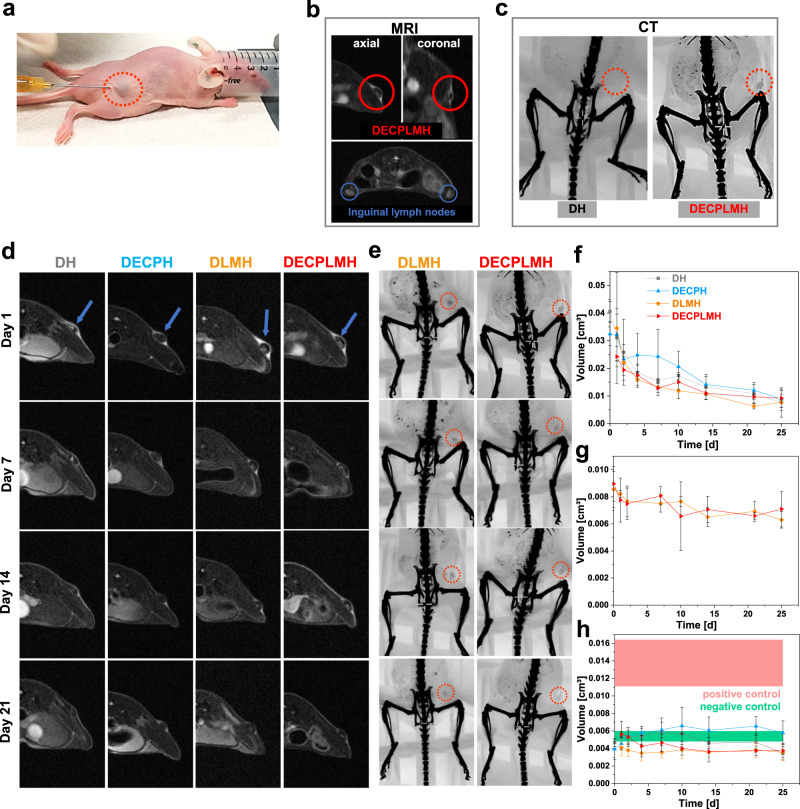
In vivo hydrogel degradation and biocompatibility. **a** Exemplary picture to show the subcutaneous injection of the DECPLMH. **b** Magnetic resonance imaging (MRI) images of hydrogels and inguinal lymph nodes. **c** Computed tomography (CT) images of hydrogels containing liquid metal nanoparticles. **d** Representative MRI and **e** CT images used for volume determination of hydrogels (red circles indicate hydrogel localization). A representative image of three individual animals is shown. **f** Volume determination of hydrogels by MRI (*n* = 3 independent animals, mean ± SD) and **g** volume of hydrogels containing liquid metal particles measured by CT (*n* = 3 independent animals, mean ± SD); **h** Inguinal lymph node size at hydrogel injection site determined by MRI, compared to negative (untreated) and positive (TPA injection) controls (*n* = 3 independent animals, mean ± SD).

To investigate the biocompatibility of the electroconductive biopolymer- and/or LM-containing materials either as implants or for topical applications, the hydrogels were injected subcutaneously in immunocompetent hairless mice (Fig. 8a and Supplementary Movie 8). An important aspect of biomaterial developments and applications is to monitor the biomaterial with methods compatible with clinical practices. Whereas the ex vivo histological analyses can illustrate the interaction between biomaterials and host tissue, in vivo imaging techniques will allow us to visualize implants in living bodies. We have previously developed MRI and infrared (IR) fluorescence imaging protocols to image injected hydrogels in mice^51^. Small animal MRI is an imaging method for characterizing tissue structures non-invasively dependent on their water content (Fig. 8b). MRI relays on the different resonance stimulation of an implant, e.g., hydrogels, and its surrounding tissue, while the difference and the resulting contrast decrease gradually during the implant degradation or integration of material into host tissue. While the two techniques provide complementary information of implants in vivo, the presence of LM in DLMH and DECPLMH hydrogels allowed the materials to be imaged with high-resolution small animal µCT.

CT scans provide non-invasive morphological evaluation of structures or tissues as different signal intensities are based on variant absorption of X-rays and are suitable for hydrogel localization and volume determination. With LM nanodroplets content as low as 0.5%, the injected DECPLMH hydrogels can be clearly visualized, whereas no visible signals can be found at the DH injection site (Fig. 8c). We used MRI and µCT to follow the in vivo degradation of the hydrogels (Fig. 8d and e). MRI shows that all the hydrogels can be degraded in the following 25 days with <30% materials left (Fig. 8f). The hydrogels have lower degradation rates in comparison to our previously studied conductive hydrogels incorporating aldehyde-modified hyaluronic acid, glycol chitosan, and a PEDOT compound, as <10% of these hydrogels were left after 12 days according to MRI measurements^38^. Interestingly, whereas the MRI signals decreased gradually over time due to matrix degradation and cell infiltration, the reduction of µCT signal was remarkably slower (with >75% material left) (Fig. 8e and g), reflecting the slow absorption of residue LM nanodroplets in vivo.

**Fig. 9:**
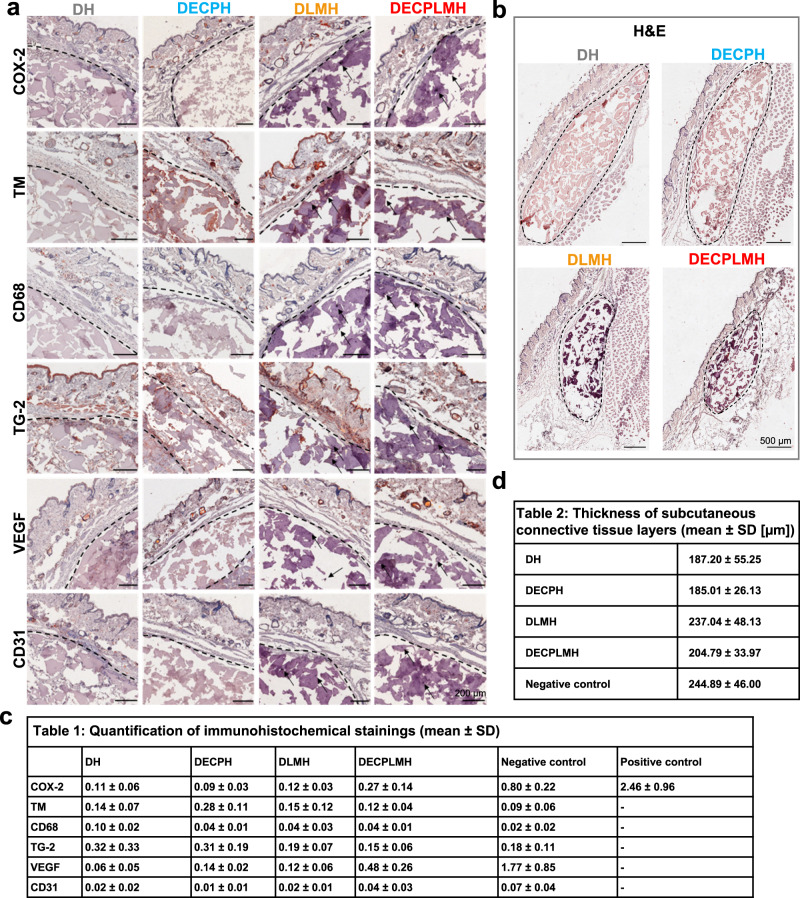
In vivo hydrogel biocompatibility. **a** Representative immunohistochemical images of markers for inflammation (COX-2, thrombomodulin TM), pan-macrophages (CD68), matrix remodeling (TG-2), and angiogenesis (VEGF, CD31), cell nuclei in blue and positive immunohistological staining in red. The black line indicates hydrogel–tissue interface and arrows point on liquid metal nanoparticles. Immunohistological stainings of three animals per group were conducted and one representative image of each group is shown. **b** Representative histological images of H&E staining. Three animals per group were conducted with H&E staining and one representative image of each group is shown. **c** Quantification of immunohistochemical stainings (positive stained area related to cell nuclei area) (*n* = 3 independent animals, mean ± SD). **d** Thickness of subcutaneous connective tissue layers based on Van Giesons’s staining compared to negative (untreated) control (*n* = 3 independent animals, mean ± SD).

MRI can also be used to evaluate foreign body reaction by measuring the volumes of inguinal lymph nodes, as swollen lymph nodes can be a sign of inflammation (Fig. 8b). For all four injectable hydrogels tested in this study, inguinal lymph node sizes were similar to the untreated (negative) control group over the entire study period. No obvious difference has been observed by comparing the lymph node sizes on the hydrogel injection site to the contralateral site (Supplementary Figs. 25 and 26). As a positive control, TPA (12-O-tetradecanoylphorbol-13-acetate) was injected and caused a remarkable increase in lymph node size (Fig. 8h). These investigations were in accordance with our previously studied conductive hydrogels^38^. The injected hydrogel adhesives caused neither local nor systemic inflammatory responses in mice. Twenty-five days after the injections, histological samples were examined. Staining of COX-2, a key inflammatory marker, was low for all mice injected with hydrogel adhesives as compared with the negative control, demonstrating that the materials did not cause adverse inflammatory reactions (Fig. 9a). Further, no significant expression of other inflammatory markers, such as thrombomodulin (TM) and CD68 (pan-macrophages), were visible for the examined hydrogels. In contrast, immunohistochemical investigation of our previously studied, fast degrading, conductive hydrogels showed a potent infiltration of macrophages reflected by positive CD68 staining^38^. The absence of macrophage infiltration in the hydrogels shown in this study could also be the reason for the slower hydrogel degradation in vivo (Fig. 9a and c).

All four injected materials do not promote matrix remodeling, vascularization, and angiogenesis, as reflected by the low staining of TG-2 (transglutaminase-2), VEGF (vascular endothelial growth factor), and blood vessel marker CD31.

For determination of the thickness of subcutaneous connective tissue layers around the hydrogel adhesives, a Van-Gieson’s staining was performed to stain elastin and collagen fibers. These connective tissue components are present and stained also in untreated mice representing the negative control, exhibiting a thickness of 245 ± 46 μm. The thickness of subcutaneous connective tissue layers around the hydrogel adhesives is in the range of 185–237 µm, being not significantly different from the negative control (Fig. 9b and d). In general, the thickness of the elastin-collagen connective tissue layer is smaller compared to gelatin-based biomaterial implants from previous investigations^52^. Moreover, a hematoxylin & eosin (H&E) overview staining of the skin samples was accomplished to analyze the morphologic organization of subcutaneous connective tissue layers, where no fibrotic alterations were observed. Together with the determination of the thickness of subcutaneous connective tissue layers, it can be concluded that no fibrous capsule was formed around the injected hydrogels. In addition, histological H&E overview staining of several organs (liver, kidney, spleen, and lymph nodes) from the biocompatibility tests in immunocompetent hairless mice with subcutaneously injected hydrogels (Supplementary Fig. 27) was performed to investigate the fates of LM and PEDOT. All organs, independent of the injected hydrogels, were not pathologically changed and we could not find any accumulated LM and PEDOT particles in the organs. Most of the LM remain at the injection site during the investigation period, as confirmed by the CT measurements, whereby minor reduction of signal intensity of the original volume and limited spread of material to peripheral area were observed. PEDOT seems to behave similarly, remaining at the subcutaneous injection site for long time. We have shown in a previous study that even after hydrogel degradation the PEDOT component was still present at the injection site, where PEDOT could be detected in the immunohistochemical staining after day 25^38^. In the future, long-term experiments should be investigated to evaluate the LM and PEDOT clearance in vivo. Taken together, the hydrogels are biologically inert as they do not cause any inflammatory or angiogenic reactions at the hydrogel–tissue interface. Imaging and histological examinations of the living animal demonstrated that the injectable adhesives can be partially degraded in vivo by host tissue in 25 days and do not cause adverse foreign body reactions.

## Discussion

Many functions of biological extracellular matrices as well as their functional versatility are associated with their complex composition. To construct such complex systems will help us to understand the underlying molecular basis of biological materials, as well as to develop smart materials. However, to introduce many different chemical functionalities into a polymer system is synthetically challenging. Herein, we developed the concept of convergent synthesis for generating complex biomatrices and designed dynamic and highly diversified reversible network including LM nanodroplets, conductive biopolymer, and functionalized polysaccharides. In this study, all precursors used in the final gelation step can be synthesized in 1–2 reaction steps and characterized, thus possible for GMP (good manufacturing practice) production for potential clinical uses. If large-scale industrial production would be required, it will be very interesting to explore further simplification of the synthesis in the future.

As a bottom-up approach, the convergent synthesis method has allowed us to exam the hypothesis that complexity and diversity of reversible chemical bonds in a self-assembled network are essential for a biomaterial to possess reliable adhesive properties. The resulting hydrogels display remarkable adhesiveness, corresponding to up to four times of clinically used fibrin glue. In the future, to apply the adhesives for utilities beyond cell culture and tissue engineering (e.g., as wearable bioelectronics), their properties at and responsiveness to other environmental conditions (e.g., drying or non-physiological temperatures) need to be investigated. The hydrogel system comprises versatile, tunable functions, including enhanced electroconductivity, fast self-healing, antibacterial activity, printability as 3D bioink, high cytocompatibility for 3D cell culture and differentiation experiments, and in vivo CT and MRI imaging applications. The injectable materials have also shown high biocompatibility in immunocompetent mice. More important, the materials do not induce an adverse host tissue response, can be partially degraded and contain the LM components over the whole experimental time frame. In the future, the modularity of this system will permit us to orchestrate the contributions of different reversible bonds by tuning individual precursors, thus lead to further improvements of adhesive and electroconductive properties, as well as promote their utilities in biomedical research and clinical applications.

## Methods

3,4-Ethylenedioxythiophene (EDOT) (Sigma-Aldrich, St. Louis, MO, USA, 483028), ammonium persulfate (APS) (Sigma-Aldrich, A3678), chitosan (Sigma-Aldrich, 448869), dextran (Sigma-Aldrich, D4876), 3,4-dihydroxyhydrocinnamic acid (Sigma-Aldrich, 102601), tannic acid (Alfa Aesar GmbH & Co KG, Karlsruhe, Germany, 36410), cholesteryl hemisuccinate (Sigma-Aldrich, C6512), eutectic gallium–indium (75.5% gallium and 24.5% indium, purity: 99.99%, Rich Metals Co., China), 4-(4,6-dimethoxy-1,3,5-triazin-2-yl)-4-methylmorpholinium chloride (DMTMM) (Sigma-Aldrich, 74104), iron(II) sulfate heptahydrate (Sigma-Aldrich, 215422), sodium (meta) periodate (Sigma-Aldrich, S1878), heparin, sodium salt, and porcine intestinal mucosa were obtained from EMD Millipore Corp., USA. PBS (phosphate-buffered saline) solution was prepared by dissolving a PBS powder in deionized water at pH 7.4. Gibco Dulbecco’s modified Eagle’s medium (DMEM, Thermo Fisher Scientific Inc., Waltham, MA, USA, 10566-016), fetal bovine serum (FBS, Sigma-Aldrich, F2442), horse serum (Sigma-Aldrich, H1270), and penicillin–streptomycin (Sigma-Aldrich, P4333), LIVE/DEAD™ Viability/Cytotoxicity Kit were obtained from Life Technologies (Carlsbad, CA, USA, L3224). C2C12 cells (C3H muscle myoblast) were purchased from ECACC and cultured in DMEM supplemented with 10% (v/v) FBS in Petri dishes in a humidified incubator at 37 °C under a 5%-CO_2_ atmosphere. We further used Cardiac Troponin T antibody (GeneTex, Inc., Irvine, CA, GTX28295), Phalloidin–Atto 633 (Sigma-Aldrich, 68825), and Hoechst 33342 NucBlue® (Life Technologies, R37605), Cell Counting Kit-8 (Dojindo EU GmbH), Click-iT™ EdU Cell Proliferation Kit for Imaging, anti-mouse Alexa Fluor™ 647 (Thermo Fisher, Invitrogen™, C10340)

### Preparation of TA-coated LM nanodroplets

Bulk EGaIn (150 mg) was added into ethanol solution (15 mL) of tannic acid (TA). The mixture was exposed to a probe sonicator (BILON92-II, Shanghai Bilon Instrument Co. Ltd, China; power of 300 W with 50% amplitude) in ice-water bath. The sonication time and tannic acid dosage were tuned to adjust the final size of EGaIn droplets. Afterward, the large droplets in the resulting suspension were removed by centrifugation at relative centrifugal force (RCF) of 123.5 × *g*, TA-coated LM nanodroplets were obtained by centrifugation at a relative centrifugal force (RCF) of 1235 × *g*, and washed with deionized water sequentially. Finally, the TA-coated LM nanodroplets were suspended in deionized water.

### Synthesis of catechol-conjugated chitosan (CHI-C)

In brief, chitosan (100 mg) was dissolved in degassed MES buffer (pH 5.0, 1 M). Hydrocaffeic acid (50 mg) and DMTMM (74 mg) dissolved in 25 mL of degassed MES buffer (1 M) were added to the chitosan solution. This mixture was kept for 12 h under nitrogen protection at room temperature. The product was purified by dialysis (Cellules Membranes, MWCO: 6–8 kD, SpectraPor, USA) against pH 5.0 HCl solution in deionized water for 72 h and only deionized water for 24 h. Samples were frozen and lyophilized at −80 °C in a freezer for 12 h. Then, the product was stored at −20 °C in a freezer before use. The synthesis of CHI-C was confirmed by using ^1^H NMR (Bruker Avance II 400 MHz; Bruker Biospin AG, Fallanden, Switzerland) (Supplementary Fig. 5) and ATR-FTIR (Bruker Optics ALPHA E spectrometer with a universal Zn Se attenuated total reflection accessory in the 400–4000 cm^−1^; Bruker Biospin AG, Fallanden, Switzerland) (Supplementary Fig. 4).

### Synthesis of cholesteryl hemisuccinate conjugated dextran (Dex-CH)

Five hundred milligrams (0.99 mmol, 1 equiv.) of dextran, 72.429 mg (0.1485 mmol, 0.15 equiv.) of cholesteryl hemisuccinate, and 48.35 mg (0.396 mmol, 0.4 equiv.) of DMAP (Dimethylaminopyridine) were added to the flask and purged with gaseous nitrogen before 25 mL of anhydrous DMSO were added to the vessel via cannulation. Then, the solution was stirred at 350 rpm for 1 h until the dextran was fully dissolved.

BOC_2_O (di-tert-butyl dicarbonate) was molten in a 37 °C-warm water bath. Then, 45.58 µL (0.41 equiv.) were added to the reaction using a plastic syringe. The reaction vessel was purged with nitrogen and stirred for 20 h at 45 °C. After the reaction mixture cooled down to RT, 10 mL of cold deionized water was added to quench the reaction and transfer the mixture to the dialysis membrane (6–8 kD). Dialysis was carried out for 5 days at room temperature while the water was changed twice per day. Then, the samples were frozen and lyophilized at −80 °C in a freezer for 12 h. Using ^1^H NMR, the product and the degree of modification were confirmed (Supplementary Fig. 7).

### Synthesis of cholesteryl hemisuccinate (CH) conjugated Dex-ALD (Dex-ALD-CH)

For the synthesis of Dex-ALD-CH, Dex-CH was first dissolved in 20 mL Milli-Q water (5% w/v, typically for 1 h). A 600-μl solution of 0.5 M sodium (meta) periodate in Milli-Q water was added dropwise to the solution of Dex-CH to a final concentration of 0.015 M. The reaction was kept for 1 h at 25 °C in the dark (as the sodium metaperiodate is light sensitive). Afterward, the reaction was stopped by the addition of glycerol (0.05% v/v) for 2 h. Then the solution of oxidized Dex-CH was dialyzed against deionized water for 72 h (molecular weight cutoff 6–8 kD) and finally lyophilized and stored at −20 °C until further processing. FTIR measurements (as described above) were performed to validate the formation of the aldehyde functional groups and confirm the successful oxidization of Dex-ALD-CH via obtained infrared spectra of the samples (Supplementary Fig. 6).

### Formation of electroconductive hydrogels

For the synthesis of the DECPLMH hydrogels, solutions of LM-TA, PEDOT:Hep and Dex-CH in Milli-Q were mixed at a volume ratio of 1:1:1 in microtubes at RT. Then, the mixture solutions were mixed with the CHI-C solutions at 1:1 volume ratio to form the DECPLMH. Hydrogels with different polymer combinations were synthesized.

### Cryo-scanning electron microscopy (cryo-SEM) and EDS

Thirty microliters droplets of freshly mixed hydrogel were placed on either onto pieces of silicon wafer (ultra-flat polished 6″-wafer with termically covered SiO_2_ film, orientation: 100, renitency: 1–50 Ohm cm^−1^, PType, endowed with boron; Plano GmbH, Wetzlar, Germany), into a perpendicularly aligned glass capillary (Thin-Wall Capillary TW150-4, 4″, 1.5-mm diameter; World Precision Instruments Germany GmbH, Friedberg, Germany), on clean normal glass slides or on cut pieces of commercially obtained pork cardiac mussels, and fixed to the cryo-SEM holder. The cryo-SEM SUPRA 40VP-31-79 (Carl Zeiss SMT Ltd., Oberkochen, Germany) equipped with an EMITECH K250X cryopreparation unit (Quorum Technologies Ltd., Ashford, Kent, UK) was used for imaging. Therefore, the hydrogel samples on the SEM holder were immediately shock-frozen in liquid nitrogen in the slushing chamber. From there they were transferred to the cryopreparation chamber at −140 °C, sublimed for 15 min at −70 °C, and sputter-coated with platinum (layer thickness 6 nm). Before sublimation and coating, several samples of pork tissue covered with hydrogel were additionally freeze-fractured inside the preparation chamber using a cold blade. Subsequently, the samples on the holder were transferred to the SEM, and then examined in a frozen state at 5 kV accelerating voltage and −100 °C temperature using the secondary electron (SE) detector. In addition, the EDS detector Bruker x-flash 6/100 and Esprit 2.0 Software (Bruker Corporation, Billerica, MA, USA), connected to the cryo-SEM, were applied to carry out energy-dispersive X-ray spectroscopy at a working distance of 4.2–10.2 mm, at 10 kV acceleration voltage, 832,942 ± 239,018 (*n* = 5) income counts per second (cps), and measurement periods of 60 s for spectra and 120 s for mapping. Thus, the presence of gallium, indium, and carbon in LM droplets was confirmed, detecting a total (element) percent by weight of 2.7 ± 0.87, 0.5 ± 0.20, and 12.2 ± 5.73, respectively (*n* = 5) (Fig. 1d).

### Transmission electron microscopy (TEM)

4-μl aliquots of liquid metal particles in ethanol were transferred to 300 mesh copper grids covered with a carbon-coated and glow-discharged formvar film. The drops were allowed to dry before they were imaged with the Jeol JEM-1400 Plus transmission electron microscope combined with the camera Ruby (JEOL, Garching, Germany) at 80 kV acceleration voltage.

In addition, hydrogel samples were dissected into small pieces washed several times in Milli-Q water and *en-bloc* contrasted with 1% uranyl acetate for 12 h at 4 °C. Then, samples were washed in bidistilled water (5 × 5 min), followed by (1) dehydration in a graded series of ethanol-water mixtures up to pure ethanol (30%, 50% 20 min each; 70%, 90%, 96% 30 min each, 3 × 100% on molecular sieve, 30 min each) and (2) infiltration into the epon substitute Embed 812 (epon-ethanol mixtures: 1:3, 1:1, 3:1 for 1 h each, followed by pure epon for 12 h, then, pure epon for 5 h). Finally, the samples were embedded into flat embedding molds, and cured at 65 °C for 12 h. Ultrathin sections (70 nm) were prepared with a Leica UC6 ultramicrotome (Leica Microsystems, Vienna, Austria), collected on formvar-coated slot grids, and stained with uranyl acetate. Contrasted ultrathin sections were analyzed with the Jeol JEM-1400 Plus TEM at 80 kV acceleration voltage.

### Strain-dependent oscillatory rheology

Rheological measurements were performed using a rheometer Anton-Paar MCR 302 (Anton-Paar GmbH, Graz, Austria). The hydrogel samples were prepared directly on the surface of the rheometer plate by first adding the mixture of components and then, adding a CH-C solution (all in PBS, pH 7.4). The plate of the rheometer was then moved down, and brought in contact with the hydrogel. Five different rheological tests were carried out: (1) The gelation process was followed by measuring the storage (G′) and loss (G″) moduli over time. (2) The stress relaxation of the hydrogels was measured over time, after an initial shear strain of 1%. (3) Amplitude and frequency sweeps were performed, where the moduli of the hydrogels were measured over a range of different strains (0.01–1000%) and frequencies (0.01–100 Hz), respectively. (4) In continuous flow measurements, the shear stress and viscosity of the hydrogels were monitored as a function of shear rate, in order to assess the shear-thinning behavior of the hydrogels. (5) Finally, the self-healing behavior of the hydrogels was assessed by measuring the G′ and G″ at alternate step-strain cycles of 1% and 1000%.

### Lap-shear tests

Lap-shear measurements were performed using the texture analyzer EZ-SX (Shimadzu Corp., Japan). The stress values were calculated by dividing the maximum shear force by the corresponding contact area created by overlapping tissue samples (400 mm^2^). Four samples of each hydrogel mixing ratio were used in the shear test (*n* = 4). Fresh vacuum-packed porcine cardiac muscle tissue was commercially obtained from the supermarket and short-time stored at 4 °C for later use. A small piece of fresh cardiac muscle was cut into rectangle sections at 20 mm × 20 mm using a razor blade. The excessive fat was shed off from the cut cardiac muscle pieces, and the thickness of test tissue sections was controlled to be 2 mm. Then, one side of cardiac muscle was fixed to a glass slide (76 mm × 26 mm) with cyanoacrylate-based superglue. The muscle tissue section on the glass slide was placed in PBS buffer to ensure tissue hydration in order during the immediate test. A droplet of polymer mixture solution was applied on a piece of muscle tissue and CHI-C was added to trigger the formation of a thin tissue-coating layer of gel, onto which the other piece of tissue was immediately brought in contact. This tissue-gel-tissue bond was pressed together, i.e., preloaded at 1.5 N in normal direction for 10 s. The overlapping area was controlled to be 20 mm × 20 mm. The samples were allowed to cure for 20 min at 25 °C. A 20-N load cell installed in the texture analyzer was used to measure lap shear of the tissue-gel-tissue bond samples, which were fixed between two film clamps to the texture analyzer and pulled apart in parallel at a velocity of 20 mm min^−1^.

### Peeling tests

A double T-peeling test was performed using the texture analyzer EZ-SX mentioned above. Therefore, two porcine cardiac tissue samples 15 mm in width (*w*), 80 mm in length, and 2 mm in thickness were stuck/joined together at the same end by the hydrogel. The overlapping area of tissue samples was 15 mm × 40 mm. The free ends of both tissue samples were clamped to the texture analyzer, respectively. Then, the upper tissue was pulled upward at a peeling angle of 90° and a constant velocity of 20 mm min^−1^ while the peeling force **F**_**p**_ was recorded. The work of adhesion required to peel the hydrogel-joined tissues apart was calculated as 2 × **F**_**p**_ w^−1^.

### Pull-off tests

Pull-off forces of different hydrogels on tissue were measured using the EZ-SX mentioned above at room temperature. Hydrogel samples having a contact area of 1.5 cm × 2.5 cm were prepared and fixed to aluminum holders of the texture analyzer using cyanoacrylate glues. They were brought in contact with a clean glass substrate and preloaded with 1.5 N for 15 min. Then, the samples were pulled-off at a speed of 20 mm min^−1^. The adhesive strength was determined by dividing the maximum force by the contact area (3.75 cm^2^).

### Electrochemical measurements

For the cyclic voltammetry tests, a three-electrode setup was used with a working electrode, a reference electrode, and a counter electrode (Supplementary Fig. 17a). A 30-μl volume of the hydrogels was formed around the working electrode, covering an electrode surface area of 0.008 cm^2^. The electrode with the hydrogel was placed inside an electrolyte solution (0.1 M PBS buffer, pH 7.4). As the potential was applied to the working electrode (as a function of the stable potential of the reference electrode) a reduction or oxidation occurred and current began to flow, which was recorded at the counter electrode. The potential that was applied at the working electrode was ramped linearly between the values −0.4 and 0.8 V and the current was measured. The current density of the hydrogels was calculated by the ratio of the measured current to the surface area of the electrode. For the impedance testing, 50-μl volumes of hydrogels were placed between two glass carbon electrodes that were connected to an electrochemistry station, i.e., the single-channel transportable potentiostat/galvanostat SP-200 (Biologic, Seyssinet-Pariset, France) (Supplementary Fig. 17b). The gap between the two electrodes was 5 mm and the diameter of the hydrogel droplet was 0.5 mm. The impedance of the hydrogels was recorded at 5 mV over a range of frequencies from 10^−2^ to 10^5^ Hz.

The areal capacitance was calculated from the voltammetry responses by summing the charge current in the positive and negative scan directions and dividing the sum by twice the scan rate, and then divide the area and mass, the areal capacitance was calculated using Eq. (1)^53^:

1 $$C=Q/(2mVUA)$$

where *C* is the areal capacitance, *m* is the mass of the gel, *V* is the voltage window, *A* is an area of the electrode, *Q* is the quantity of electricity, and *U* is the range of potential.

### Conductivity testing

We used the 2-probe testing method to test the conductive of wet bulk gel. In order to exclude the effects of the ionic conductivity, the gels were formed with Milli-Q-water-dissolved LM-TA, CHI-C, Dex-ALD-CH, and conductive polymers.

As shown in Supplementary Fig. 17b, we first realized the impedance testing, for what 50-μl volumes of hydrogels were placed between two glass carbon electrodes that were connected to the electrochemistry station mentioned above. The gap between the two electrodes was 5 mm, and the diameter of hydrogel droplets was 0.5 mm. The impedance of the hydrogels was recorded at 5 mV over a range of frequencies from 10^−2^ to 10^5^ Hz. The conductivity (*σ*) of the hydrogels was calculated using Eq. (2)^54,55^:

2 $$\sigma =\frac{{{\mathrm{Re}}}\left(Z\right)}{\left[{{\mathrm{Re}}}\left(Z\right)\right]2+\left[{{\mathrm{Im}}}\left(Z\right)\right]2}\times \frac{d}{S}$$

where Re(*Z*) and Im(*Z*) are the real and imaginary parts of the impedance (*Z*) at a frequency of 1 Hz, which is comparable to frequencies in physiological tissues^56^. *d* is the diameter and *S* is the area of the electrode.

### 3D printing

The bioprinting tests were performed by an in-house custom-built 3D printer (Supplementary Fig. 16). This printer had a custom linear guide, allowing the use of syringes as print heads. The hydrogels (0.1% LM-TA, 1% CHI-C, 1% Dex-ALD-CH, 1% PEDOT:Hep) were preformed inside the syringe and extruded during the printing process. As a proof of principle a 3 layers scaffold was printed (Fig. 4g). The 3D printer was controlled by the Repetier-Host software and the 3D models (STL-files) were converted to code (G-Code) by the software Slic3r (http://slic3r.org/).

### Determination of antibacterial activity of hydrogels

DH, DECPH, DLMH, and DECPLMH hydrogels were selected as the experimental groups for antibacterial tests, while the blank served as the contrastive group. Four parallel samples of each group were used. Both *Bacillus subtilis* (Ehrenberg) Cohn (Bacillales, Bacillaceae) and *Escherichia coli* (Migula) Castellani & Chalmers (Enterobacterales, Enterobacteriaceae) were obtained from Prof. Dr. Thorsten Mascher (Technische Universität Dresden, Germany) and Dr. Adrian Keller (Paderborn University, Germany) and cultured in Luria-Bertani (LB) broth for 12 h prior to the experiment. 1 mL of each bacterial solution was added to 4 mL of fresh LB media in clean test tubes and allowed for additional culturing for 3 h. The hydrogel samples were placed in a 96-well plate and incubated to full formation at 37 °C for 1 h. Then, 100 µL of fresh LB broth was applied to rinse the hydrogel. Concentrations of bacterial solution were measured and then diluted 5 times with fresh LB broth. Subsequently, 100 µL of each diluted bacterial suspension (10^6^ CFU mL^−1^) was added to the surface of each hydrogel sample. The hydrogel covered with the bacterial solutions was incubated for 12 h at 37 °C. Afterward, 50 µL of bacterial suspension on each sample were collected and transferred to a 96-well plate for optical density measurement (OD) at 600 nm with an MQX200 spectrophotometer (BioTek Instruments Inc., Winooski, VT, USA).

The bactericidal ratio of the hydrogels was calculated according to Eq. (3):

3 $${\mathrm{Bactericidal}}\,{\mathrm{ratio}}\,( \% )=\frac{{\mathrm{OD}}\,{\mathrm{of}}\,{\mathrm{contrastive}}\,{\mathrm{group}}\mbox{-}{\mathrm{OD}}\,{\mathrm{of}}\,{\mathrm{experimental}}\,{\mathrm{group}}}{{\mathrm{OD}}\,{\mathrm{of}}\,{\mathrm{control}}\,{\mathrm{group}}}\times 100 \%$$

### Culturing of C2C12, MSCs, and L929 cells

A vial of frozen C2C12 cell line (ECACC), MSCs (provided from Prof. Martin Bornhaeuser, Dresden Stem Cell Lab, Department of Internal Medicine I, University Hospital Carl Gustav Carus, Technische Universität Dresden) or L929 cells (ECACC) was thawed in a 37 °C water bath for 2 min. The cells were transferred to the 5-mL full cell culture medium (DMEM GlutaMax low glucose with 10% FBS), after mixing, the suspension was transferred into a 15 mL falcon tube and recollected. The supernatant was discarded and the cell pellet suspended in 7-mL full cell culture medium. The cell suspension was transferred to a T-25 cell culture flask and incubated at 37 °C, 95% relative humidity under a 5%-CO_2_ atmosphere. To preserve the myoblast characteristics, the passage of the cells was performed at cell confluency between 50 and 60%.

### Culturing cells in the hydrogel

10 µL CHI-C (contains 2 × 10^3^ cells) and 10 µL polymer blend were subsequently mixed in an uncoated angiogenesis µ-slide (81506, Ibidi GmbH, Gräfelfing, Germany), incubated at 37 °C for 20 min, washed two times with DMEM medium (10% FBS, 10 µL), covered with DMEM medium (10% FBS, 20 µL), and kept in the incubator at 37 °C under a 5%-CO_2_ atmosphere for specific time point.

### Live-dead cell staining

The cells were seeded into the hydrogel in the µ-slide mentioned above and DMEM medium (10% FBS, 10 µL) was added. 24, 72, and 168 h after the initial setup of culturing, the resulting ~2 µM calcein-AM and 4 µM EthD-1 working solution was added directly to cells and incubated at 37 °C for 1 h. Then, the wells with samples were observed under a confocal laser scanning microscope Zeiss LSM 780 (Carl Zeiss Microscopy GmbH, Jena, Germany) and the pictures were exported by ZEN 3.0 (Carl Zeiss Microscopy GmbH, Jena, Germany)

### Differentiation of C2C12 cells and immunofluorescence staining

C2C12 cells were cultured in the hydrogels at a cell density of 3000 cells per well in the above-mentioned µ-slide. When cells achieved 50% confluence, the 10% FBS DMEM medium was replaced with low-glucose DMEM supplemented with 2.5% horse serum and 1% penicillin/streptomycin in order to induce cell differentiation. Then, 72 and 168 h after the initial setup of culture in differentiation medium, the cells were fixed with 4% PFA for 1 h. Cells were then permeabilized with 0.1% Triton X-100 (Sigma-Aldrich, T9284) for 20 min at 25 °C, followed by blocking with 3% BSA for 12 h at 4 °C. Subsequently, cells were incubated with phalloidin for 30 min or exposed to immunofluorescent staining. Therefore, samples were incubated with primary mouse anti-Cardiac Troponin T antibodies (Abcam, ab8295) diluted at a ratio of 1:400 for 2 h. Secondary antibodies, anti-mouse Alexa Fluor 647 (Invitrogen, A-21235) were applied at a ratio of 1:500 for 1 h and nuclei were counterstained with Hoechst 33342 (1:4000). Then, samples were imaged with the confocal laser scanning microscope mentioned above.

### In vitro L929 and MSCs proliferation tests

For quantification of cell proliferation in vitro, 30 µl hydrogels were first formed in the wells of 96-well plates, then L929 cells (5 × 10^2^) and MSCs (5 × 10^2^) were seeded in a 96-well plate incubated in DMEM containing 10% (v/v) fetal bovine serum (FBS) and 1 % (v/v) penicillin–streptomycin at 37 ◦C. After different culturing time (1st day, 7th day, 14th day, and 21st day), the proliferation was calculated using CCK-8 assay.

### EdU (5-ethynyl-2′-deoxyuridine) staining and assay

The MSCs or L929 were co-culture with DH and DECLMH in 96-well plates. After 1, 7, 14, and 21 days’ culturing, the EdU (5-ethynyl-2′-deoxyuridine) was added to the cell culture medium, the cells then incubated at 37 °C for 6 h with EdU containing medium. The cells were then fixed by using paraformaldehyde solution (4%), followed by a 0.5% Triton^TM^ X-100 permeabilization treatment for 10 min. Remove the permeabilization buffer, then wash the cells in each well twice with 100 µl of 3% BSA in PBS. After removing the wash solution, 50 µl of Click-iT reaction cocktail was added to each well, incubate the plate for 2 h at room temperature, protected from light. After removing the reaction cocktail, then washing each well once with 100 µl of 3% BSA in PBS. Adding 50 µl of 1X Hoechst 33342 (5 µg mL^−1^ solution) per well. Incubate for 30 min at room temperature, protected from light. Observe the wells via inverted Laser scanning microscopy (Zeiss LSM 780, Jena, Germany). Twelve images per sample were captured. ImageJ was used to quantify the number of EdU+ and Hoechst+ cells. Proliferation activity was defined as the percentage of EdU+ cells in relation to the total number of Hoechst+ cells.

### In vivo biocompatibility testing

#### Hydrogel injection

Animal experiments were conducted in accordance with the guidelines of German Regulations for Animal Welfare. The local Ethical Committee for Animal Experiments (“Landesdirektion Sachsen”) approved the underlying protocol (reference number DD24.1-5131/450/16) and the animal experiments were conducted at the Helmholtz-Zentrum Dresden-Rossendorf (Institute of Radiopharmaceutical Cancer Research). Hydrogels were gelated for 5 min before injection and injected as described previously^51^. Briefly, female immunocompetent hairless SKH1-Elite mice were purchased from Charles River and housed at 27 ± 1 °C and 55 ± 5% humidity with 12 h light cycle. Mice (age 8–9 weeks, weight 20–25 g) were anesthetized using 8% (v/v) desflurane (Baxter) and subcutaneously injected with 50 µl hydrogel (either DH, DECPH, DLMH, and DECPLMH) in the lower back area (Fig. 8a).

### In vivo hydrogel volume measurements

Hydrogel volume and inguinal lymph node size were measured using dedicated 7T small animal magnetic resonance imaging (MRI, Bruker) and ParaVision software with a T2-weighted measuring sequence (TRARE) (Fig. 8b and d). Echo and repetition time was 38 ms and 5774 ms, respectively. Spatial resolution was 150 µm in xy-direction and a slice thickness of 0.8 mm was applied. In addition, CT measurements (50 keV, 630 µA, and a 300 ms exposure time) were conducted to determine the volume of hydrogels with incorporated liquid metal particles using the nanoScan^®^ PET/CT (Mediso) and Nucline NanoScan software. Quantification of the hydrogel volume and lymph node size was performed using the software ROVER (v3.0.57 h, ABX GmbH).

### Histological analysis

Endpoint histological analysis was performed at day 25 after injection of the hydrogels as described previously^57^. Briefly, three animals per group were sacrificed, and the remaining hydrogels with the surrounding tissue were removed. Tissue samples were fixed in 4% (v/v) PFA for 24 h and, hereinafter, in 20% (w/v) sucrose in PBS for 3 d at room temperature. For cryosection, samples were bisected in the middle of the remaining hydrogel and embedded in PBS solution containing 7.5% (w/v) gelatin and 20% (w/v) sucrose. After freezing, samples were cut to 5-µm sections in a cryostat at −30 °C. Hematoxylin & eosin (H&E) stain and Van-Gieson’s stain to measure thickness of subcutaneous connective tissue layers were performed as described previously using standard protocols^52^. Regarding the measurement of the thickness of subcutaneous connective tissue layers, for each section, 5 points on each site (skin and muscle site) of the remaining hydrogel were measured using AxioVision software (Carl Zeiss). In addition, specific tissue response was visualized using immunohistochemical stainings for cyclooxygenase-2 (COX-2, inflammation), TM (inflammation), CD68 (pan-macrophages), transglutaminase-2 (TG-2, matrix remodeling), vascular endothelial growth factor (VEGF, angiogenesis), and CD31 (blood vessel) as described previously^57^. After antigen retrieval in 10 mM heated citrate buffer in four heating cycles (except for CD68), quenching of endogenous peroxidase in 3% (v/v) hydrogen peroxide for 10 min and quenching of endogenous biotin using Biotin-Blocking System from Dako according to manufacturer’s instructions was performed. Unspecific binding was blocked by incubation in 10% (v/v) FCS in PBS for 1 h before tissue sections were incubated with primary antibody (anti-COX-2, ab15191, Abcam, 1.500, rabbit; anti-TM, sc9162, Santa Cruz Biotechnology, 1:50, rabbit; anti-CD68, MCA-1957, AbD Serotec, 1:100, rat; anti-TG-2, sc20621, Santa Cruz Biotechnology, 1:50, rabbit; anti-VEGF, sc152, Santa Cruz Biotechnology, 1:100, rabbit; anti-CD31, ab28364, Abcam, 1:75, rabbit) or isotype control (normal rat IgG, sc-2026, Santa Cruz Biotechnology; rabbit polyclonal IgG, ab27478, Abcam; concentrations comparable to primary antibody) at 4 °C for 12 h. Sections were incubated with biotinylated secondary antibody against rabbit (IgG-biotinylated, 111-065-003, Dianova, 1:200, goat) or rat (IgG-biotinylated, 312-066-045, Dianova, 1:200, rabbit) for 1 h and visualized afterward by incubation with ExtrAvidin peroxidase (Sigma-Aldrich) for 30 min and AEC substrate kit (BD Biosciences) for 2–5 min. Sections were counterstained with Mayer’s hematoxylin and embedded in aqueous solution. Sections were imaged using AxioImager.A1 microscope and AxioVision software (version 4.8, Carl Zeiss). Quantification of immunohistological stainings was performed using ImageJ/FIJI (version 1.52i). Therefore, a color threshold plugin was used and RGB values were set for cell nuclei and immunohistochemical positively stained areas. After applying the analyze particles plugin, positively stained area was divided by the cell nuclei area.

Statistical significance of hydrogel and inguinal lymph node volume over the time course, as well as for immunohistochemical investigations was calculated using a two- or one-way ANOVA, respectively, followed by a Bonferroni post hoc test using Prism 7 (GraphPad Software). No statistical significant differences in hydrogel volume, lymph node size or immunohistochemically stained targets were found.

### Statistical analysis

Unless otherwise noted, all experiments were performed in triplicate (*n* = 3) and data are presented as mean ± s.d. Statistical analysis was carried out using the software OriginPro 2017 (OriginLab Corp.), and applying the one-way ANOVA followed by Tukey multiple pairwise comparison tests. *P*-values <0.05 were considered as statistically significant.

### Reporting summary

Further information on research design is available in the Nature Research Reporting Summary linked to this article.

## Supplementary information

Supplementary Information

Description of Additional Supplementary Files

Supplementary Movie 1

Supplementary Movie 2

Supplementary Movie 3

Supplementary Movie 4

Supplementary Movie 5

Supplementary Movie 6

Supplementary Movie 7

Supplementary Movie 8

Reporting Summary

## Data Availability

All data supporting the results in this study are available within the article and its supplementary information or from the corresponding author upon reasonable request.
