# Supplementary material for: Convergent synthesis of diversified reversible network leads to liquid metal-containing conductive hydrogel adhesives
Source: Nat Commun. 2021 Apr 23;12:2407. doi: 10.1038/s41467-021-22675-2 (PMC8065207; doi:10.1038/s41467-021-22675-2)
Supplement: Supplementary file 1 — Supplementary Information [file 41467_2021_22675_MOESM1_ESM.pdf]

Supplemental Information

Convergent Synthesis of Diversified Reversible Network Leads to Liquid Metal-Containing Conductive Hydrogel Adhesives

Yong Xu[1], Rebecca Rothe[2,3], Dagmar Voigt[4], Sandra Hauser[3], Meiying Cui[1], Takuya Miyagawa[1], Michelle Patino Gaillez[1], Thomas Kurth[5], Martin Bornhäuser[6,7], Jens Pietzsch[2,3*] and Yixin Zhang[1,8*]

1 Technische Universität Dresden, B CUBE Center for Molecular Bioengineering, 01307 Dresden, Germany.

2 Technische Universität Dresden, School of Science, Faculty of Chemistry and Food Chemistry, 01062 Dresden, Germany.

3 Helmholtz-Zentrum Dresden-Rossendorf (HZDR), Institute of Radiopharmaceutical Cancer Research Department of Radiopharmaceutical and Chemical Biology, 01328 Dresden, Germany.

4 Technische Universität Dresden, Institute for Botany, Faculty of Biology, 01062 Dresden, Germany.

5 Technische Universität Dresden, Center for Molecular and Cellular Bioengineering (CMCB), Technology Platform, EM Facilty, Fetscherstraße 105, 01307 Dresden, Germany.

6 Center for Regenerative Therapies Dresden (CRTD), Technische Universität Dresden, Fetscherstraße 105, 01307, Dresden, Germany.

7 University Hospital Carl Gustav Carus der Technischen Universität Dresden, Medizinische Klinik und Poliklinik I, Fetscherstraße 74, 01307, Dresden, Germany.

8 Cluster of Excellence Physics of Life, Technische Universität Dresden, 01062 Dresden, Germany.

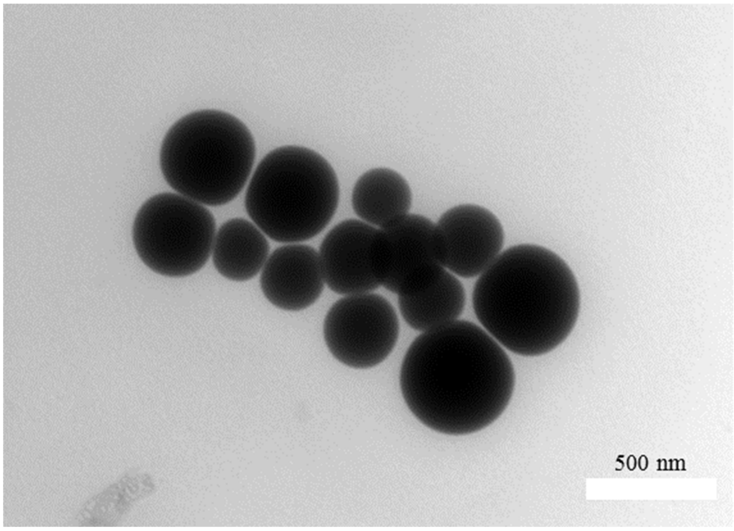

Supplementary Fig. 1: TEM image of LM nanodroplets without TA coting, scale bar = 500 nm. A representative image of three individual experiments is shown.

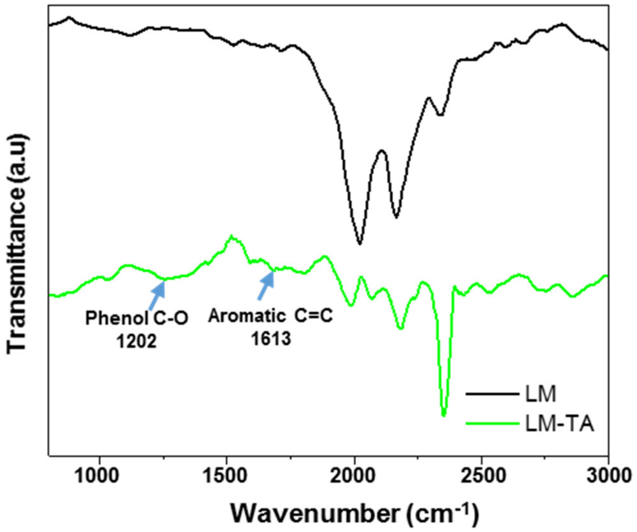

Supplementary Fig. 2: Fourier Transform Infrared (FT-IR) spectra of LM, LM-TA. Characteristic vibration of the phenol group in TA at 1202 cm^{-1} (ν C–O) and the aromatic group in TA at 1613 cm^{-1} (ν C=C) in LM-TA nanodroplet.

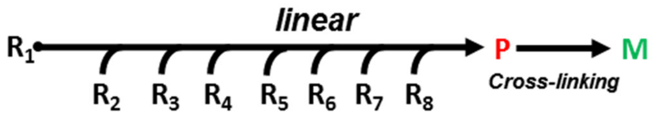

Supplementary Fig. 3: Schematic illustration of polymer synthesis with linear synthesis strategy.

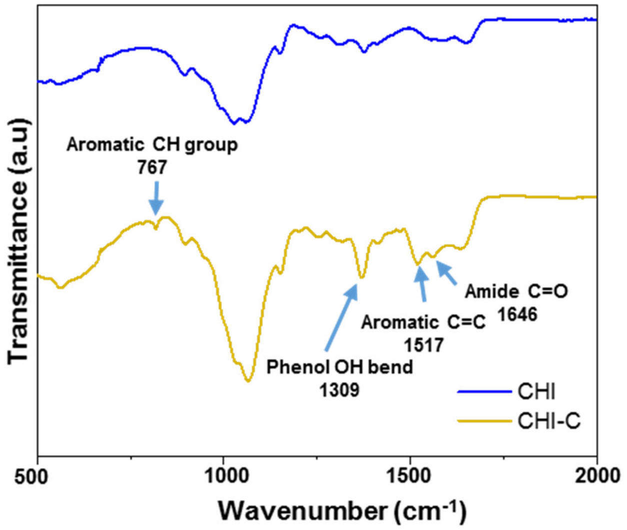

Supplementary Fig. 4: Fourier transform infrared (FT-IR) spectra of CHI, CHI-C. The FTIR spectrum showed the appearance of the characteristic peaks for the amide C=O (~1646 cm^{-1}), aromatic C=C (~1517 cm^{-1}) and CH bends (~767 cm^{-1}), phenol OH bend (~1309 cm^{-1}) which indicates the hydrocaffeic acid conjugation to chitosan.

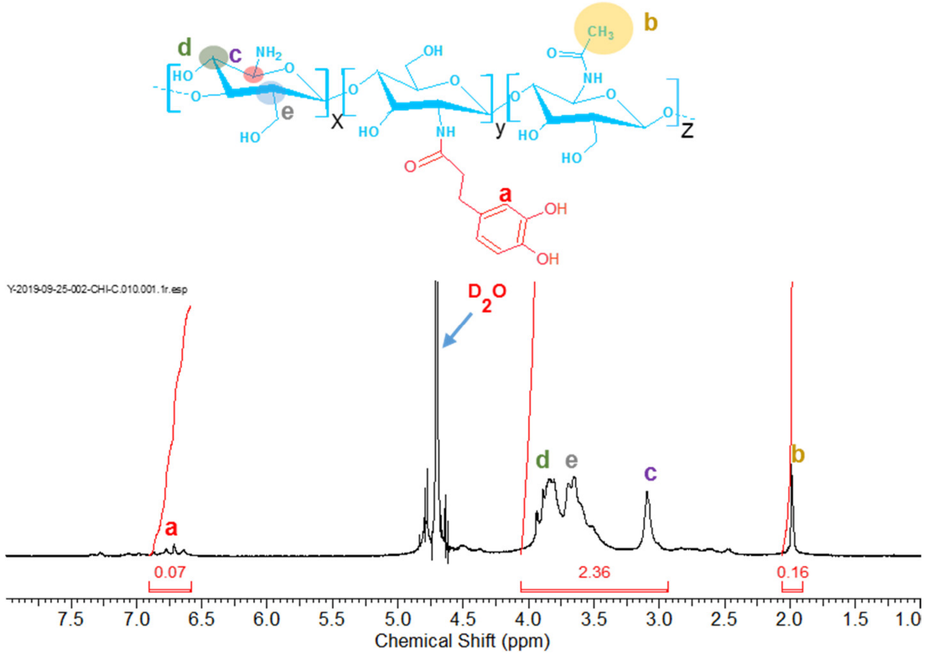

Supplementary Fig. 5: Proton nuclear magnetic resonance (^{1}H-NMR) spectra of chitosan-hydrocaffeic acid (CHI-C). The hydrocaffeic acid modified chitosan has a resonance at 2.0 ppm corresponding to the methyl protons of the acetylated glucosamine residues, peaks between 3.0 and 4.0 ppm assignable to the protons of C-3, C-4, C-5, and C-6 of the glucosamine residues. In the case of the hydrocaffeic acid-modified chitosan, new peaks in between 6.5 and 7.0 ppm which indicate the proton in aromatic ring are assignable to hydrocaffeic acid are observed, that confirms the conjugation of hydrocaffeic acid to the chitosan backbone.

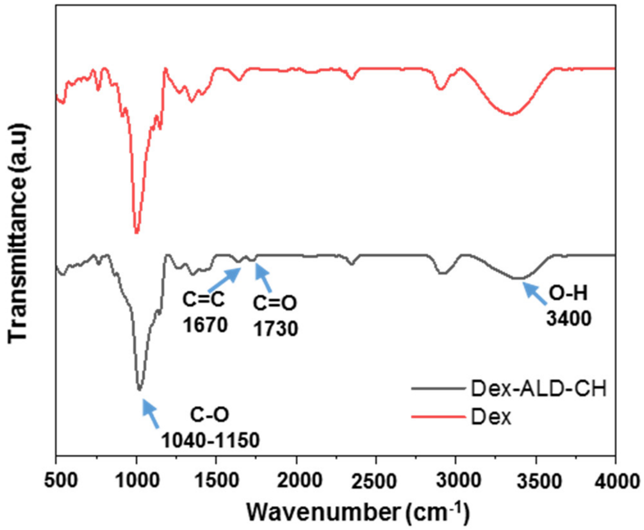

Supplementary Fig. 6: Fourier transform infrared (FT-IR) spectra of Dex, Dex-ALD-CH. The FTIR spectrum showed the peak at 1730 cm^{-1} that corresponds to the aldehyde groups in the Dex-ALD-CH sample, C=C(~1670 cm^{-1}) which indicates the cholesterol hemisuccinate is conjugated to Dextran backbone.

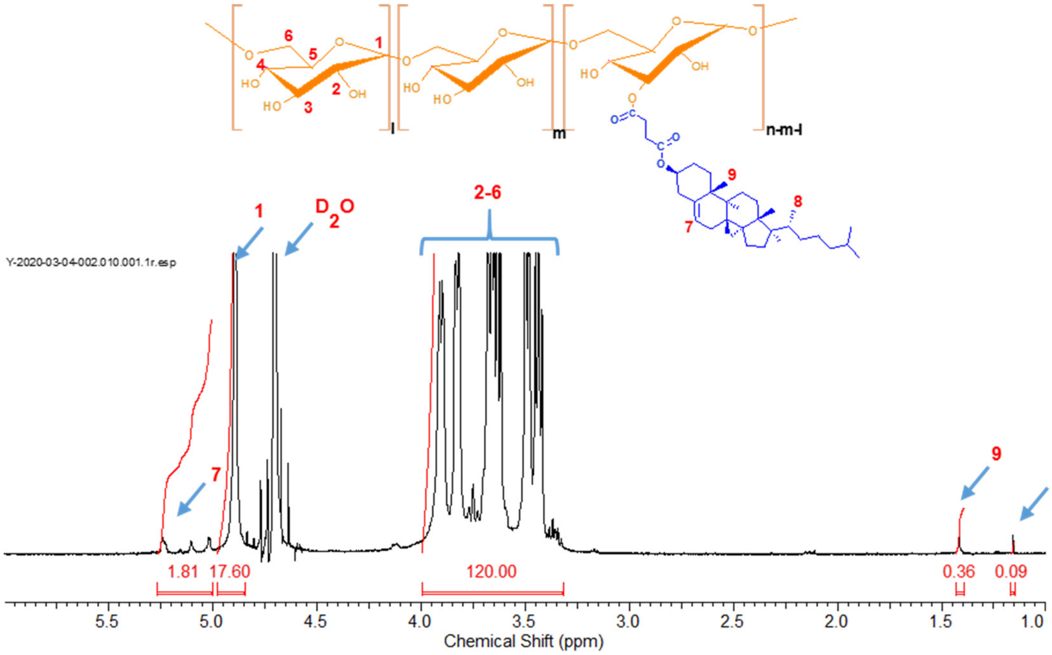

Supplementary Fig. 7: Proton nuclear magnetic resonance (^{1}H-NMR) spectra of Dextran-cholesteryl succinate (Dex-CH). In the case of the cholesteryl succinate-modified Dex has a resonance at 4.95 ppm corresponding to the the anomeric proton (H1) in C1 position, protons (H2-H6) at the C2-C6 positions were detected at 3.52-4.02 ppm. Peaks in 5.28 ppm, 1.2 ppm and 1.42 ppm which indicate the proton in cholesteryl hemisuccinate are observed, that confirm the conjugation of cholesterol succinate to the Dextran backbone.

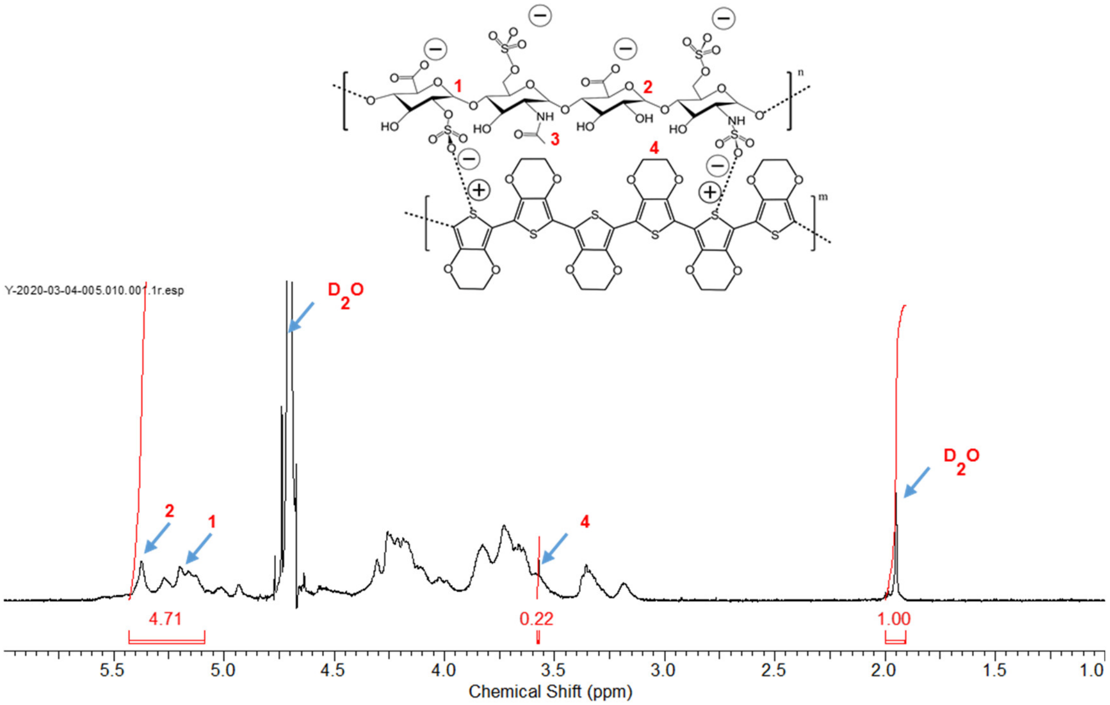

Supplementary Fig. 8: Proton nuclear magnetic resonance (^{1}H-NMR) spectra of heparin and PEDOT:Hep. The signals of 1, 2 indicate anomeric protons of heparin and 3 indicates the acetyl group of N-acetyl-D-glucosamine in the molecular structure of heparin, 4 indicates the -O-CH_2- protons of EDOT units.

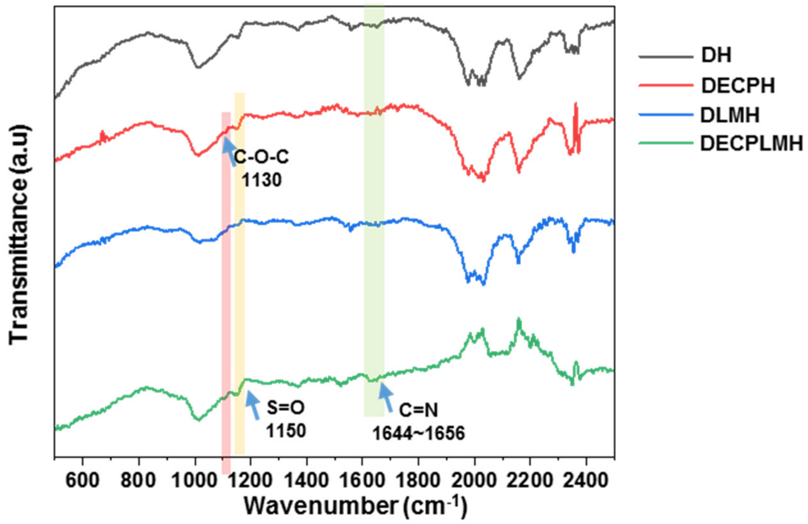

Supplementary Fig. 9: Fourier transform infrared (FT-IR) spectra of DH, DECPH, DLMH and DECPLMH. The FTIR spectrum showed the peak at 1644 cm^{-1}~1656 cm^{-1} corresponding to the Schiff base (-C=N-), which is formed between the aldehyde groups of Dex-ALD-CH and the amine groups of CHI-C. S=O (~1150 cm^{-1}) and C-O-C (~1130 cm^{-1}) indicate the incorporation of the PEDOT:Hep in the DECPH and DECPLMH.

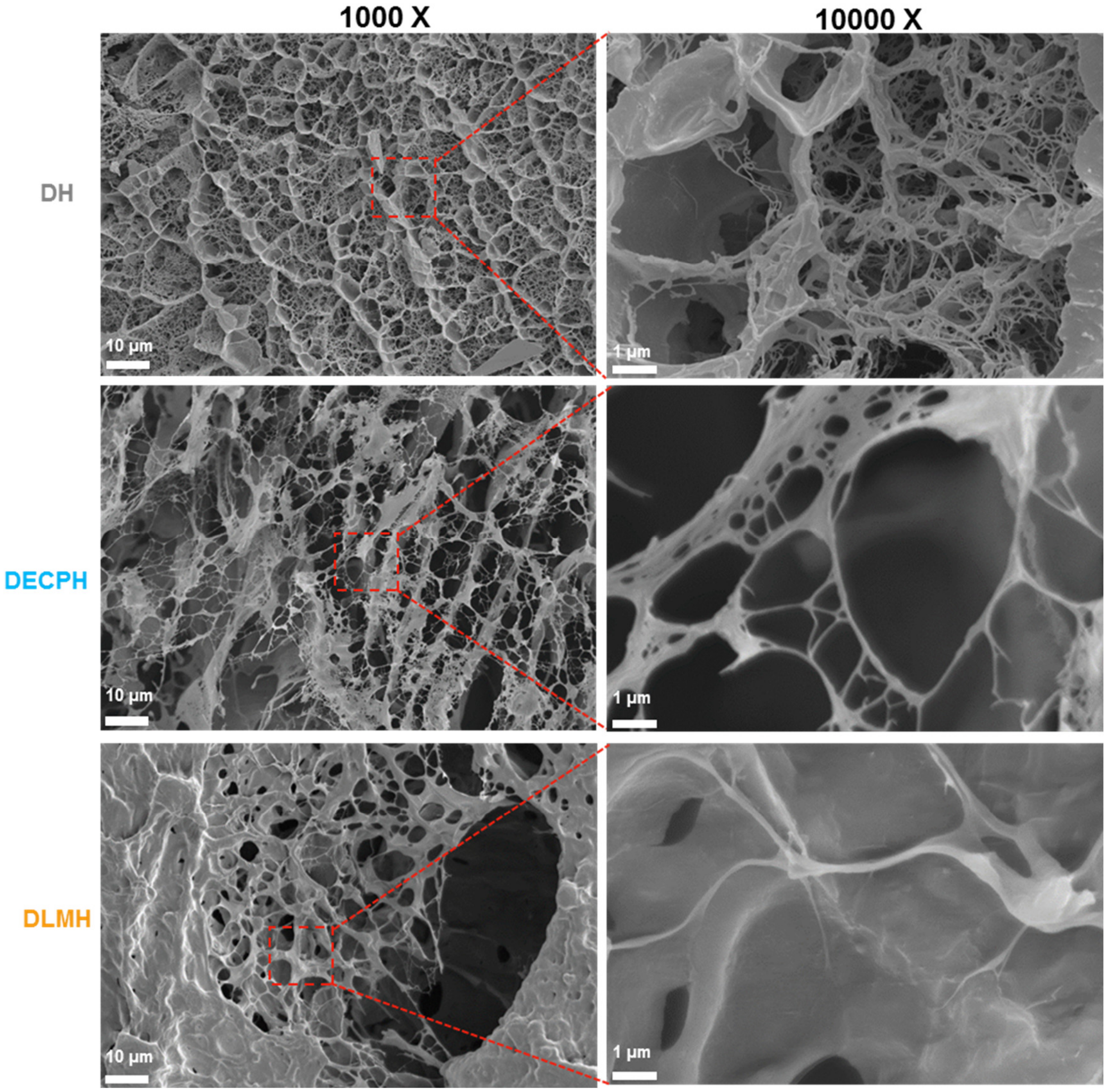

Supplementary Fig. 10: Cryo-SEM images of hydrogel networks of DH, DECPH, DLMH. A representative image of three individual samples is shown.

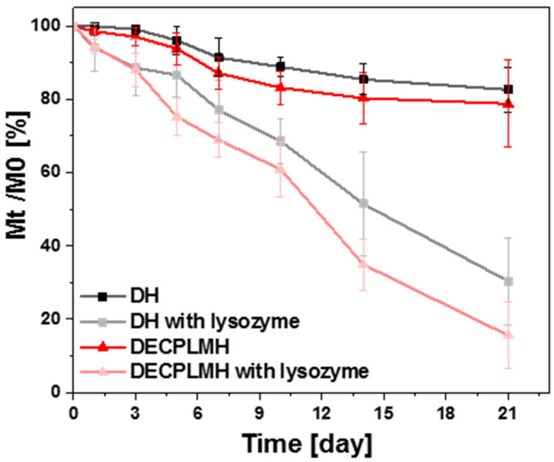

Supplementary Fig. 11: Degradation of hydrogels in PBS with and without lysozyme, M0 represents the weight of original as prepared hydrogels and Mt represents the weight of hydrogels at specific time point ((n=4 independent hydrogels, mean ± SD).

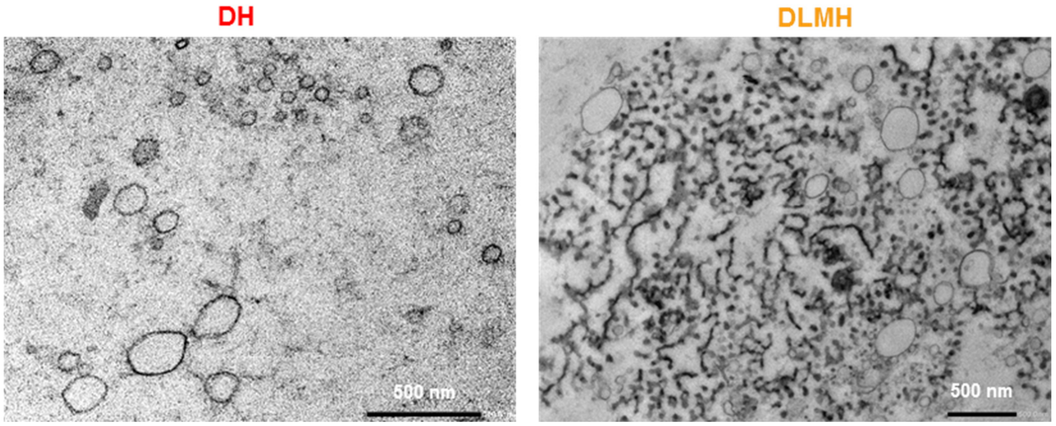

Supplementary Fig. 12: Transmission electron microscopy (TEM) images of hydrogel networks of DH, DLMH. A representative image of three individual experiments is shown.

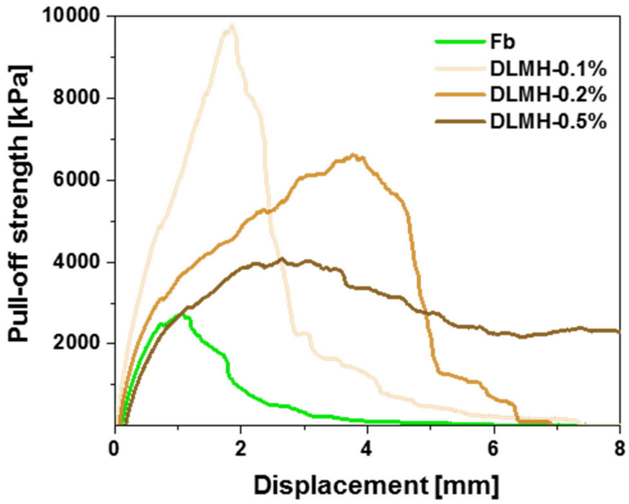

Supplementary Fig. 13: Adhesion pull-off testing of hydrogels, where the adhesive strength is plotted against the extension (~distance).Inset: Detail of the experimental setup.

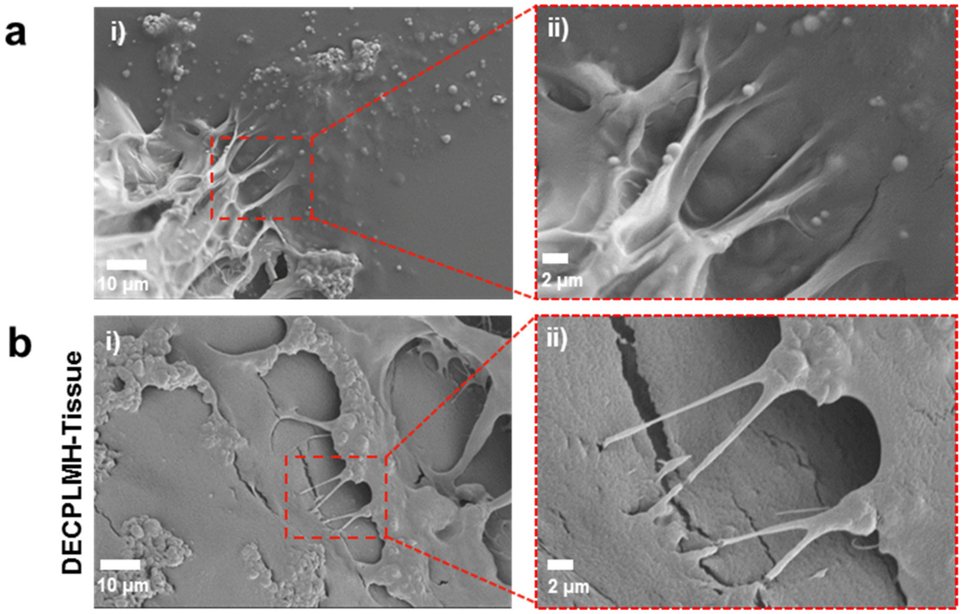

Supplementary Fig. 14: Cryo-Scanning electron microscopy (cryo-SEM) images of (A) the DECPLMH-glass interface, (B) the DECPLMH-tissue interface; overview (i) and detail (ii). A representative image of three individual experiments is shown.

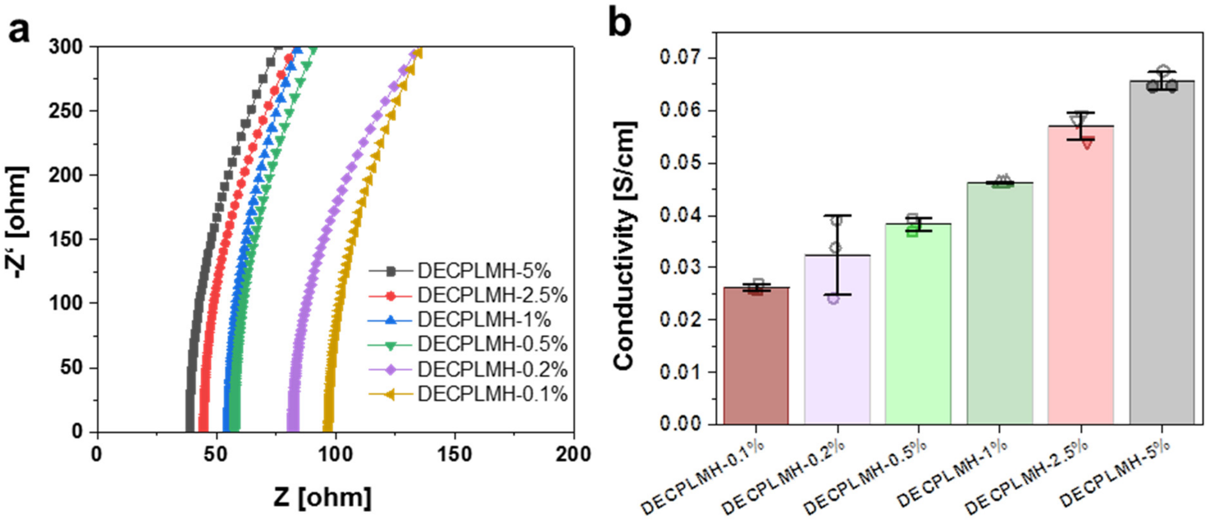

Supplementary Fig. 15: Electrochemical impedance spectroscopy of DECPLMH with different concentration (W/V) of LM nanodroplets. **a** Nyquist plot of the DECPLMH hydrogels. **b** The conductivity of DECPLMH hydrogels. n = 3 individual hydrogel samples, mean ± SD.

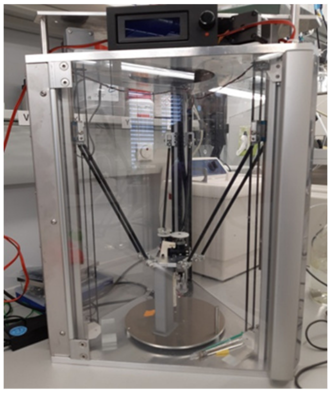

Supplementary Fig. 16: The image of the custom-made 3D printer.

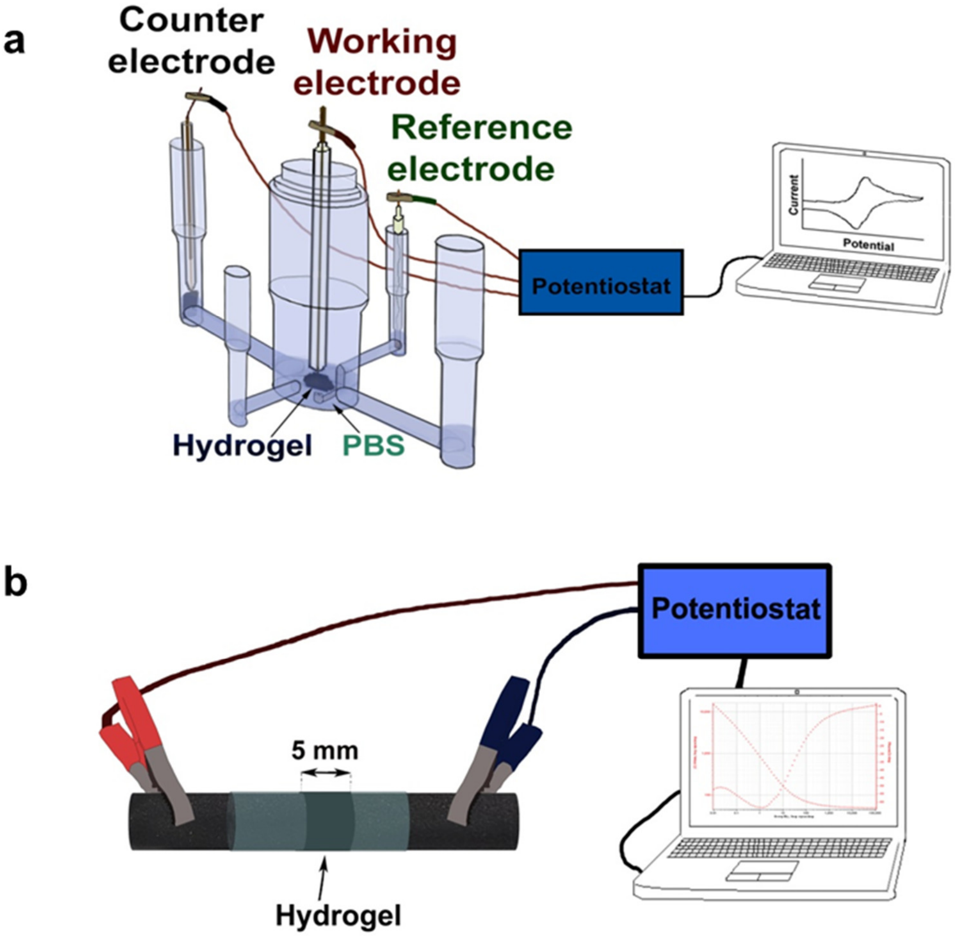

Supplementary Fig. 17: **a** Scheme of the experimental setup of the cyclic voltammetry tests. **b** Scheme of the experimental setup of the impedance measurements.

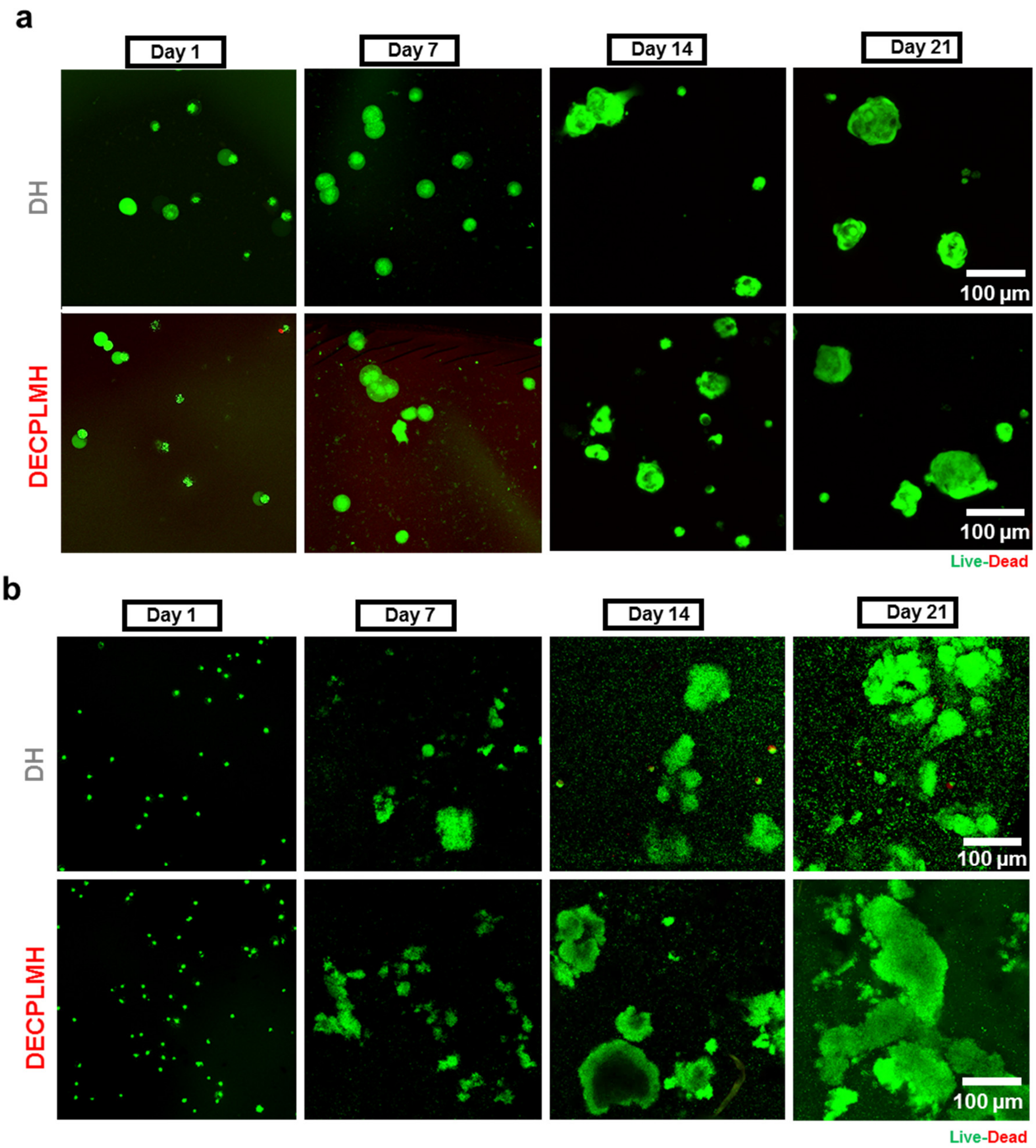

Supplementary Fig. 18: Biocompatibility of the DH and DECPLMH *in vitro*. a Live-dead staining of MSCs cultured in hydrogels at day 1, day 7, day 14 and day 21 with calcein-AM (Green; Viable) and ethidium homodimer-1 (Red; Dead). Scale bar: 100 µm. A representative image of three biological replicates is shown. **b** Live-dead staining of L929 cells cultured in hydrogels at day 3 and day 7, with calcein-AM (Green; Viable) and Ethidium homodimer-1 (Red; Dead). Scale bar: 100 µm. A representative image of three biological replicates is shown.

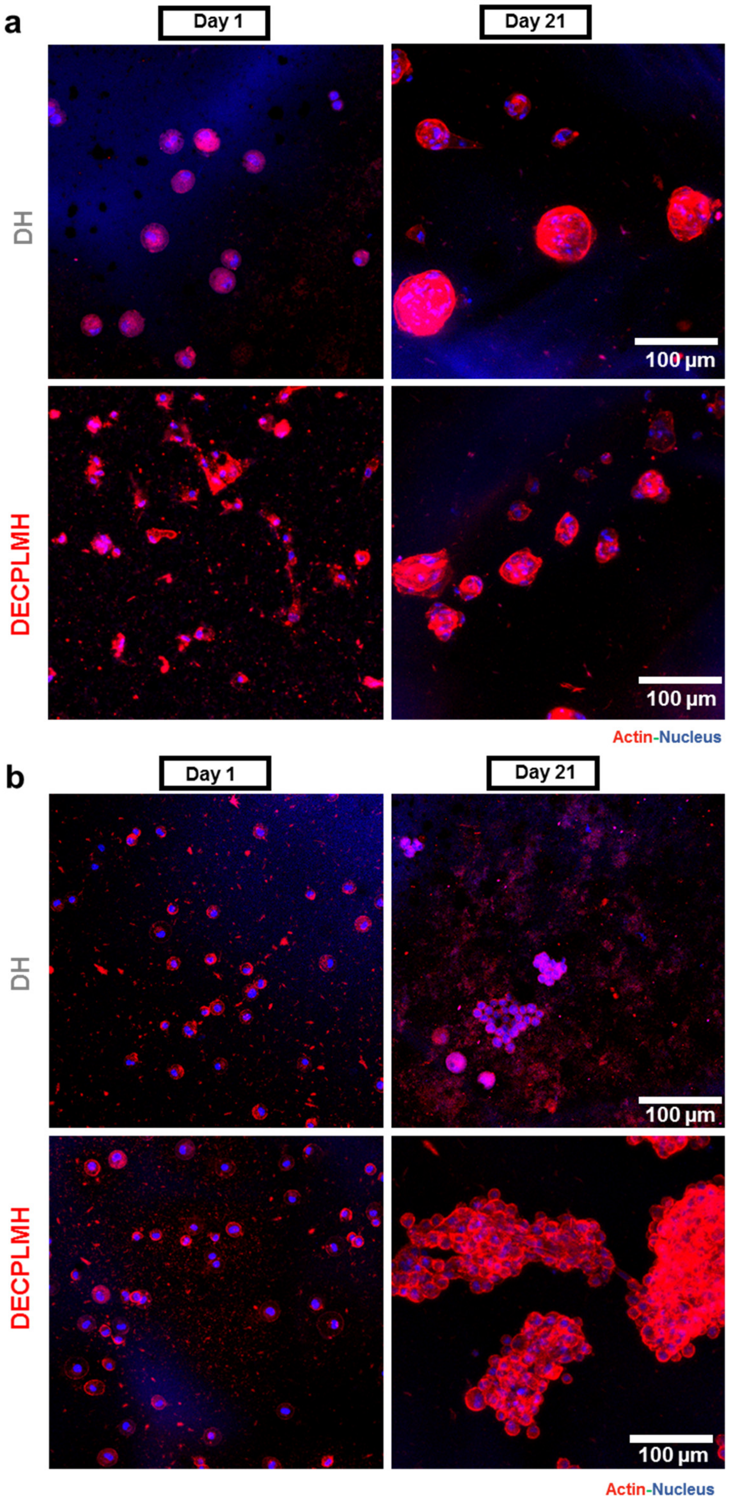

Supplementary Fig. 19: (A) Fluorescence staining of the MSCs cultured in DH and DECPLMH at day 1, day 21 of the nuclei (Blue; Hoechst) and actin (Red; Phalloidin). Scale bar: 100 µm. A representative image of three biological replicates is shown (B) Fluorescence staining of the MSCs cultured in DH and DECPLMH at day 1, day 21 of the cell nucleus (Blue; Hoechst) and actin (Red; Phalloidin). Scale bar: 100 µm. A representative image of three biological replicates is shown.

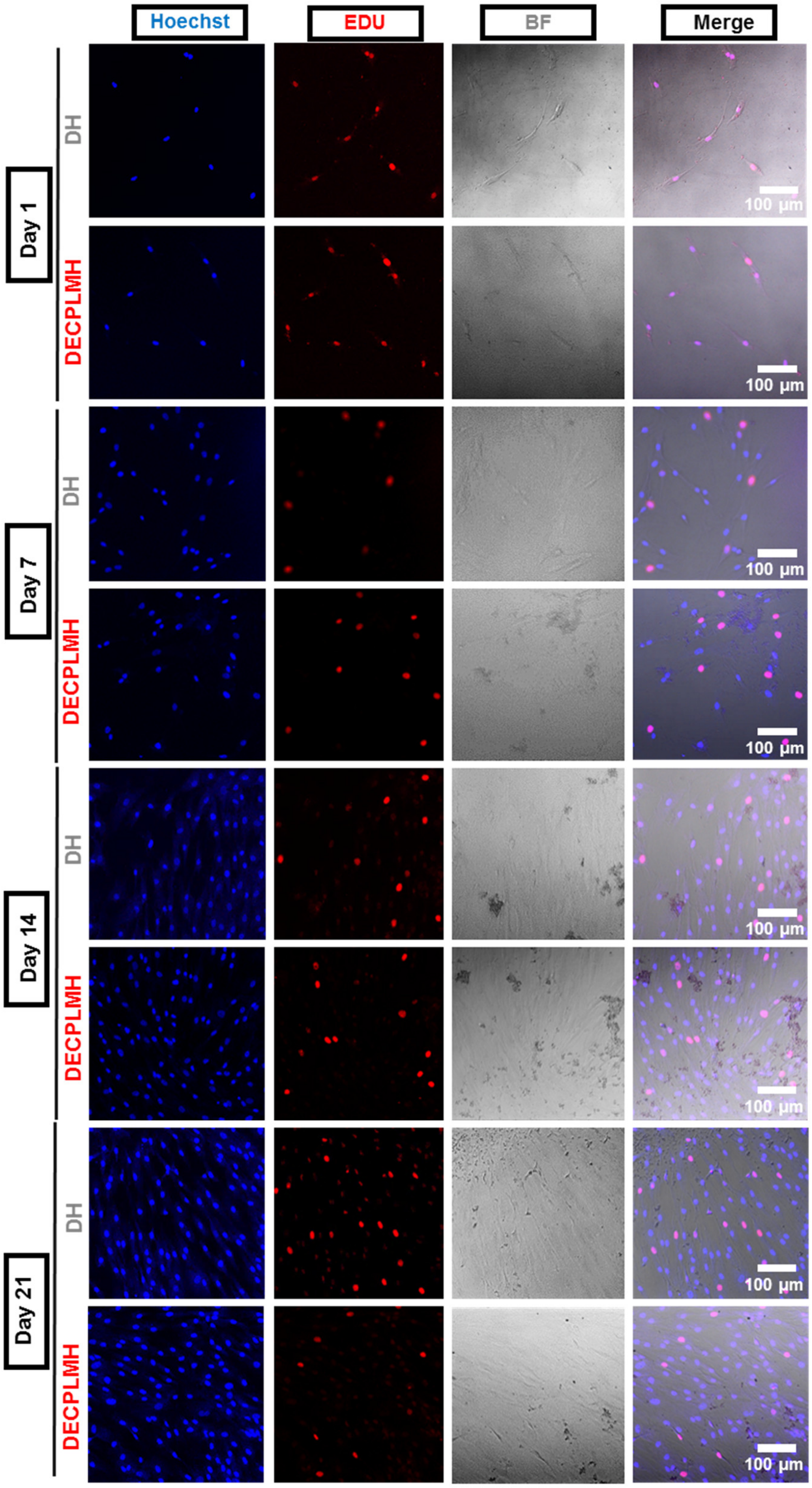

Supplementary Fig. 20: EdU (Red) and Hoechst (Blue) staining of MSCs in 2D co-culture with DH and DECPLMH at day 1, 7, 14, and 21. Scale bar: 100 μm. A representative image of four biological replicates is shown.

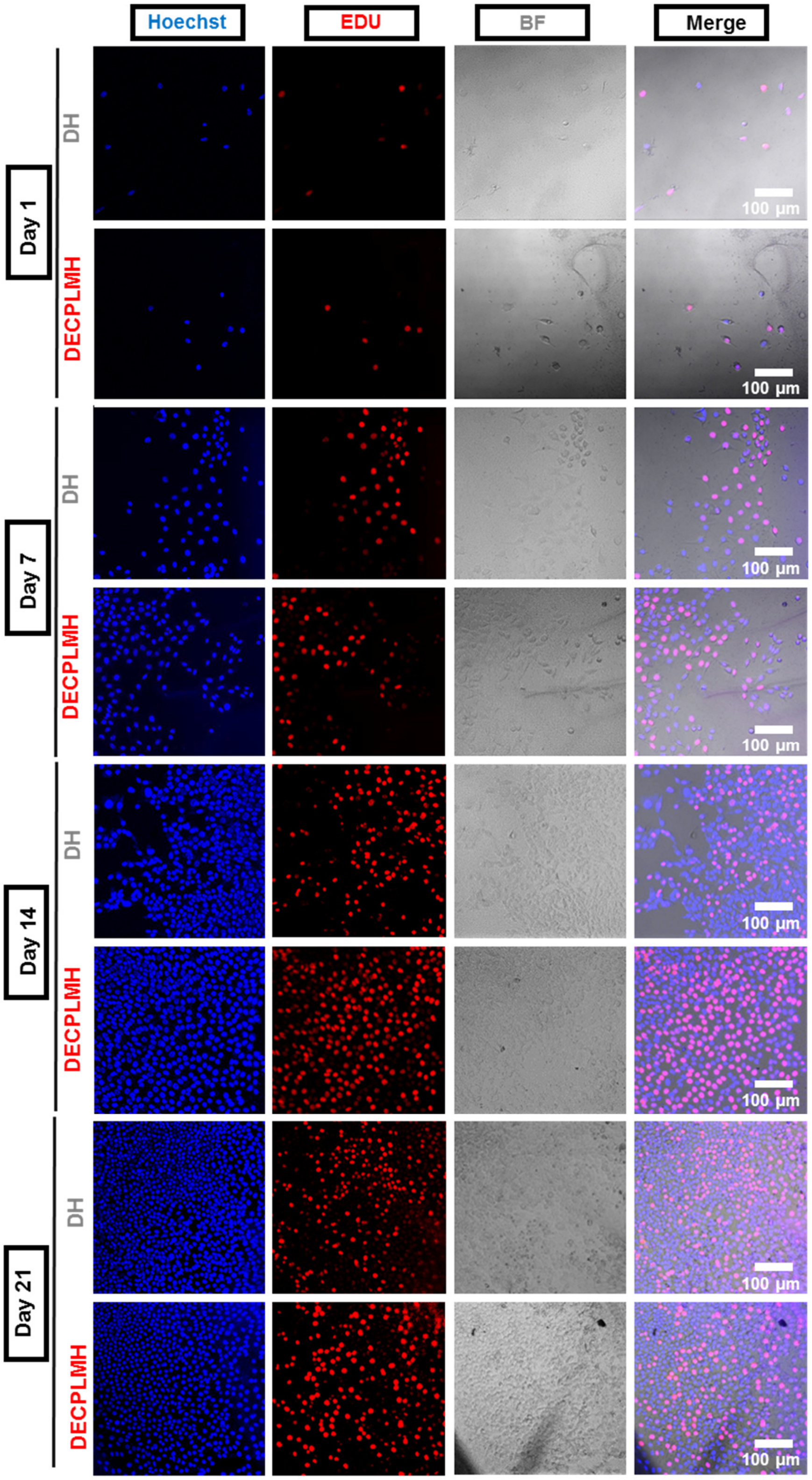

Supplementary Fig. 21: EdU (Red) and Hoechst (Blue) staining of L929 cells in 2D co-culture with DH and DECPLMH at day 1, 7, 14, and 21. Scale bar: 100 μm. A representative image of four biological replicates is shown.

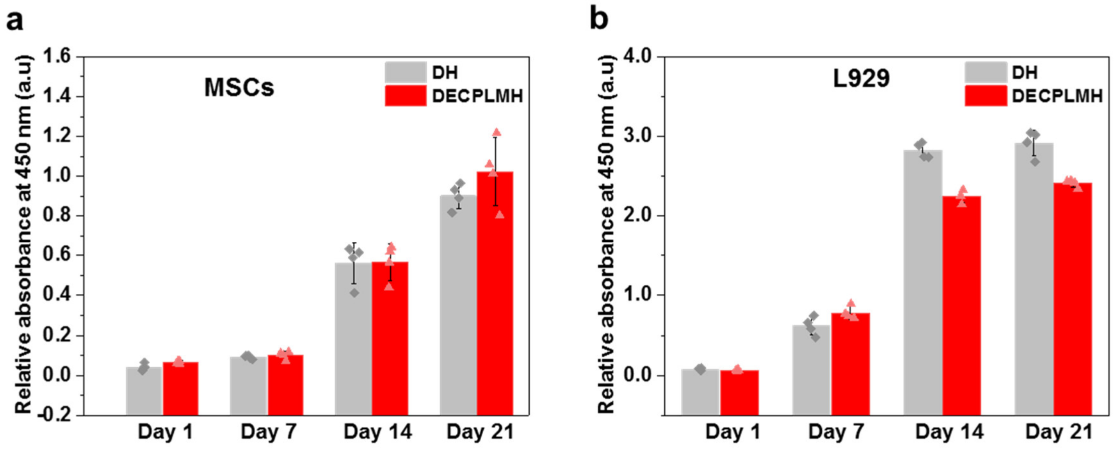

Supplementary Fig. 22: Proliferation assay of **a** MSCs and **b** L929 cells in 2D co-culture with DH and DECPLMH via CCk-8 tests at day 1, 7, 14, and 21. (n=4 biologically independent samples, data are presented as means ± SD)

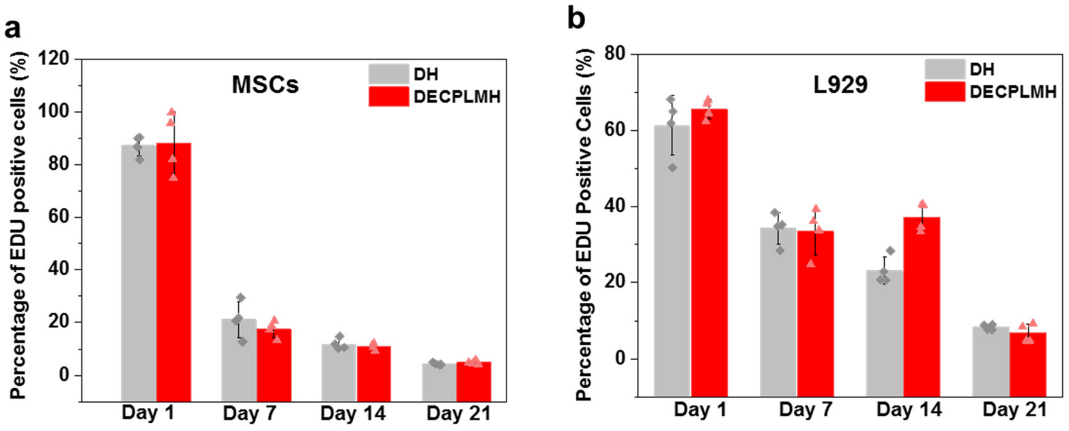

Supplementary Fig. 23: EdU assay of **a** MSCs and **b** L929 cells in 2D co-culture with DH and DECPLMH on day 1, 7, 14, and 21. Proliferation activity defined as the percentage of EdU+ cells in relation to the total number of Hoechst+ cells on day 1, 7, 14 and 21. (n=4 biologically independent samples, data are presented as means ± SD).

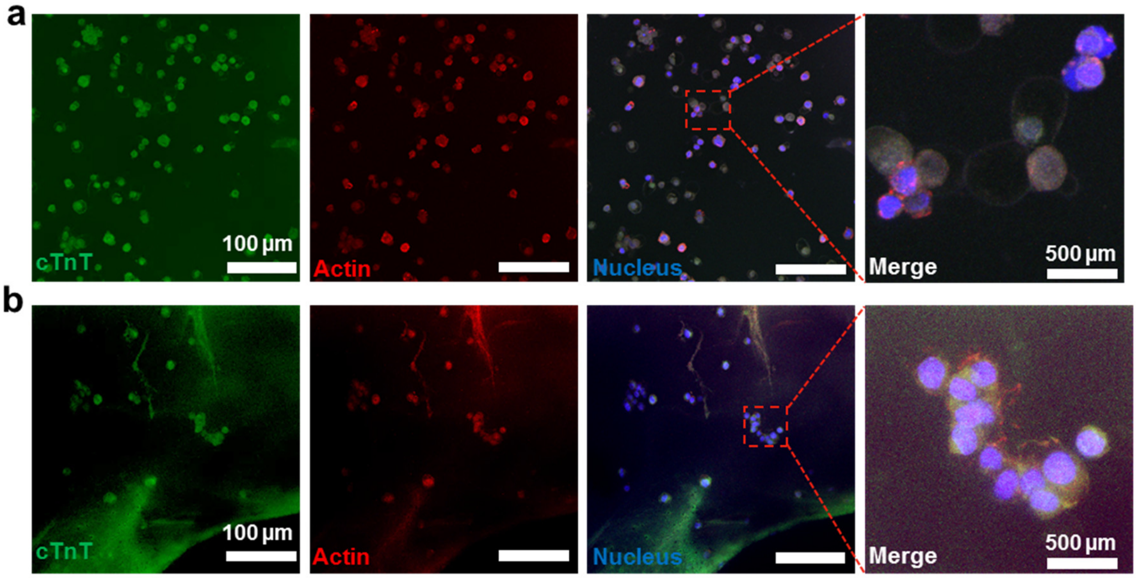

Supplementary Fig. 24: cTnT (Green) and actin (Red) immunofluorescence staining of C2C12 cells cultured in **a** DH and **b** DECPLMH hydrogels with differentiation medium for 10 days. Scale bar: 100 µm. A representative image of three biological replicates is shown.

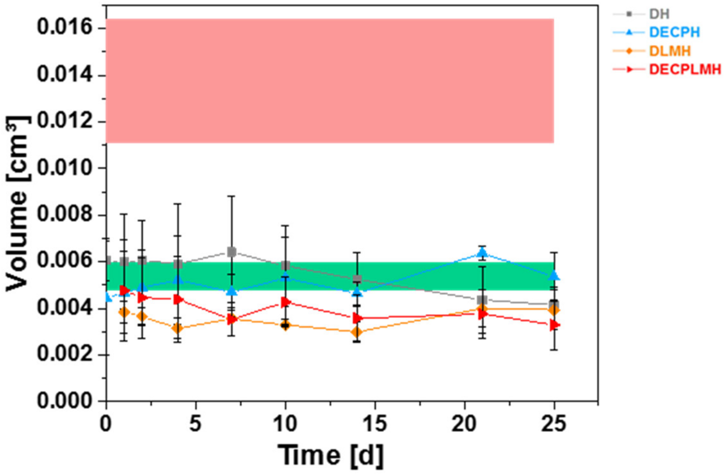

Supplementary Fig. 25: Inguinal lymph node size at contralateral site determined by MRI, compared to negative (untreated) and positive (TPA injection) controls. (n=3 independent animals, data are presented as means ± SD).

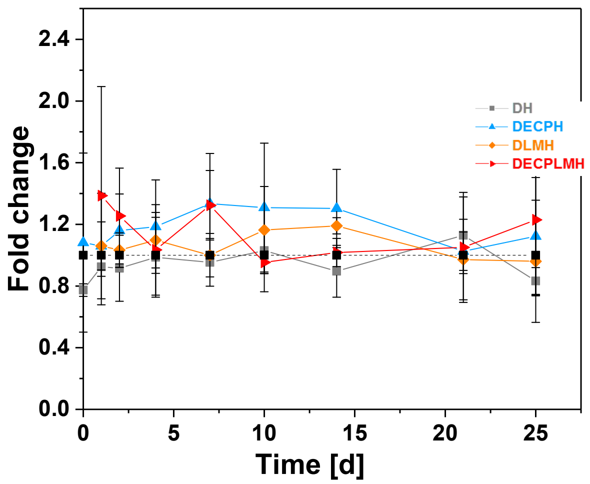

Supplementary Fig. 26: Ratio of lymph node size at the injection site compared to site without hydrogel injection. (n=3, biologically independent animals, data are presented as means ± SD).

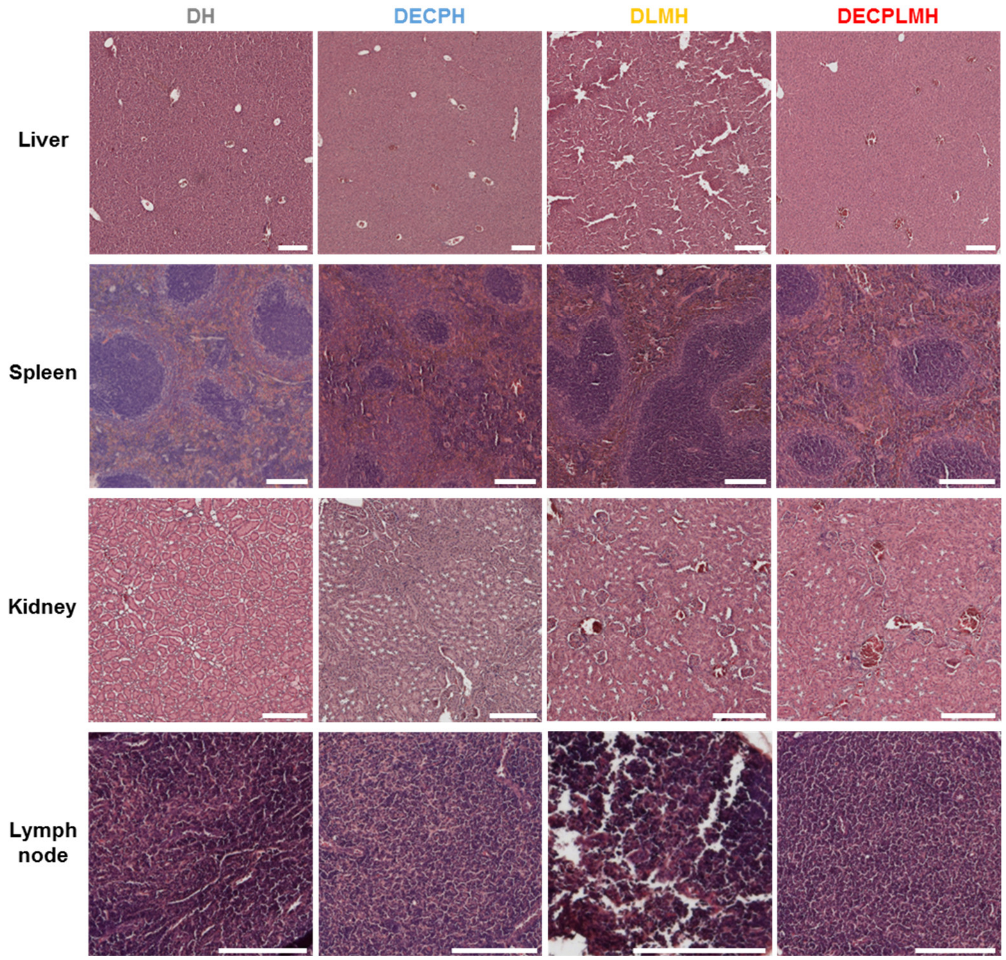

Supplementary Fig. 27: H&E overview staining of the organs (liver, kidney, spleen and lymph nodes) from the biocompatibility test with injected hydrogels. Scale bar = 200 µm. 3 animals per group were conducted with H&E staining and one representative image of each group is shown.

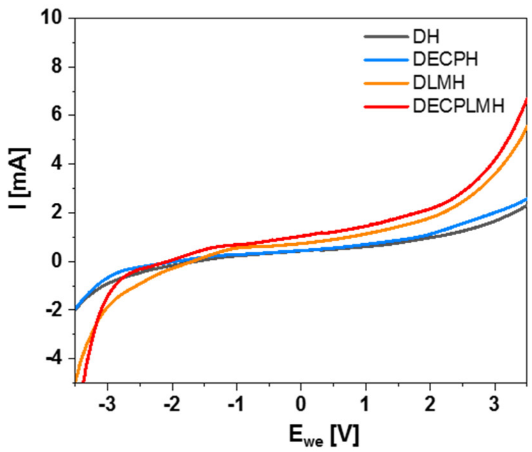

Supplementary Fig. 28: Current-voltage (I–V) curves of hydrogels with the range of voltage from -3.5 to +3.5V.

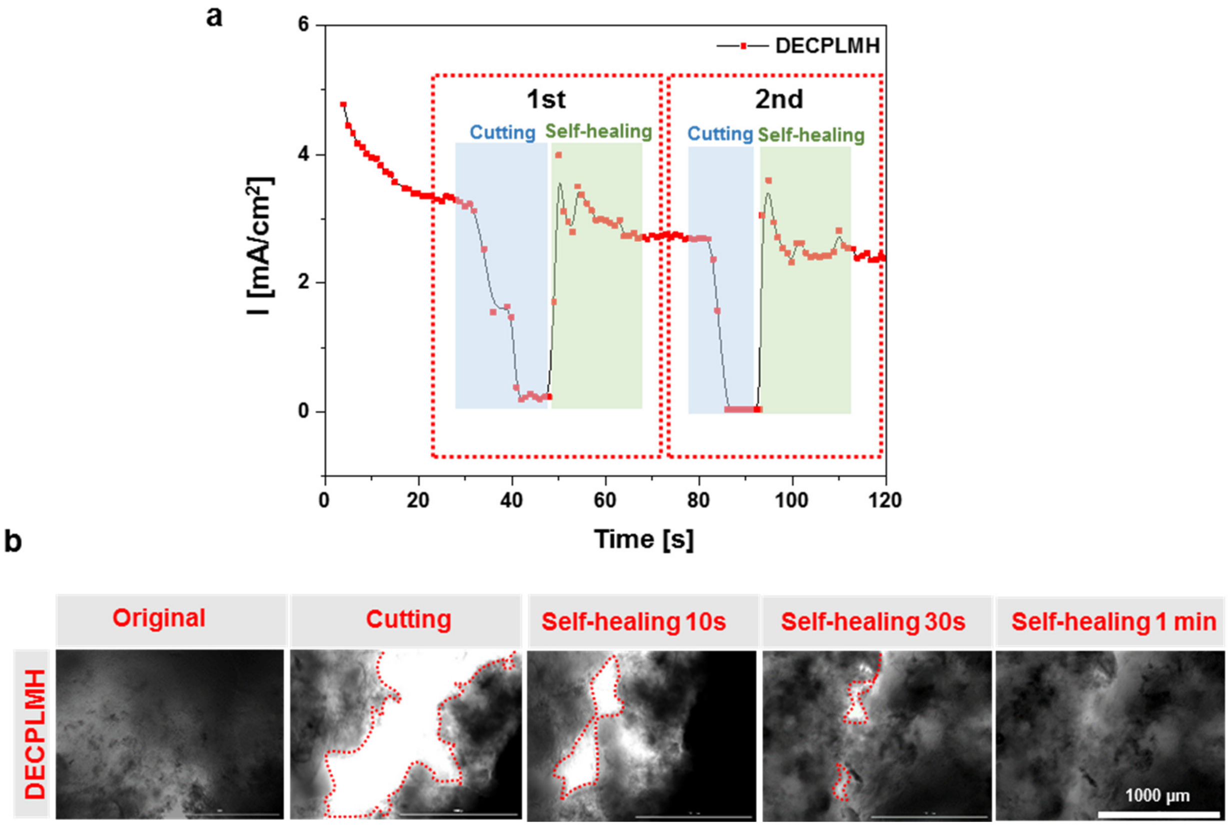

Supplementary Fig. 29: a Current response of DECPLMH during two cycles' cutting and self-healing process. The current decreased to zero under coverslip cutting process but slowly recurred because of the hydrogel's self-healing capability after removing the coverslip, which means the conductivity of the hydrogel also recovered. **b** Bright filed images of the self-healing process of DECPLMH hydrogel. A representative image of three individual hydrogel is shown.
