# Supplementary material for: Convergent synthesis of diversified reversible network leads to liquid metal-containing conductive hydrogel adhesives
Source: Nat Commun. 2021 Apr 23;12:2407. doi: 10.1038/s41467-021-22675-2 (PMC8065207; doi:10.1038/s41467-021-22675-2)
Supplement: Supplementary file 2 — Description of Additional Supplementary Files [file 41467_2021_22675_MOESM2_ESM.docx]

**Description of Additional Supplementary Files**

**Supplementary Movie 1:** Hydrogel was injected on the glass dish.

**Supplementary Movie 2:** Hydrogel stacked to the tweezer after self-healing process.

**Supplementary Movie 3:** Hydrogel was injected into PBS solution.

**Supplementary Movie 4:** Hydrogel 3D printing on the cell culture dish.

**Supplementary Movie 5:** Hydrogel 3D printing on the porcine skin.

**Supplementary Movie 6:** PBS treatment after 3D printing on the cell culture dish.

**Supplementary Movie 7:** PBS immersion after hydrogel 3D printing on the porcine skin.

**Supplementary Movie 8:** The subcutaneous injection of the hydrogel.
