# Supplementary material for: Convergent synthesis of diversified reversible network leads to liquid metal-containing conductive hydrogel adhesives
Source: Nat Commun. 2021 Apr 23;12:2407. doi: 10.1038/s41467-021-22675-2 (PMC8065207; doi:10.1038/s41467-021-22675-2)
Supplement: Supplementary file 11 — Reporting Summary [file 41467_2021_22675_MOESM11_ESM.pdf]

nature research

Corresponding author(s): Yixin Zhang

Last updated by author(s): Feb 17, 2021

Reporting Summary

Nature Research wishes to improve the reproducibility of the work that we publish. This form provides structure for consistency and transparency in reporting. For further information on Nature Research policies, see our Editorial Policies and the Editorial Policy Checklist.

Statistics

For all statistical analyses, confirm that the following items are present in the figure legend, table legend, main text, or Methods section.

n/a	Confirmed	
☐	☒	The exact sample size (n) for each experimental group/condition, given as a discrete number and unit of measurement
☐	☒	A statement on whether measurements were taken from distinct samples or whether the same sample was measured repeatedly
☐	☒	The statistical test(s) used AND whether they are one- or two-sided<br>*Only common tests should be described solely by name; describe more complex techniques in the Methods section.*
☐	☒	A description of all covariates tested
☐	☒	A description of any assumptions or corrections, such as tests of normality and adjustment for multiple comparisons
☐	☒	A full description of the statistical parameters including central tendency (e.g. means) or other basic estimates (e.g. regression coefficient) AND variation (e.g. standard deviation) or associated estimates of uncertainty (e.g. confidence intervals)
☐	☒	For null hypothesis testing, the test statistic (e.g. F, t, r) with confidence intervals, effect sizes, degrees of freedom and P value noted<br>*Give P values as exact values whenever suitable.*
☒	☐	For Bayesian analysis, information on the choice of priors and Markov chain Monte Carlo settings
☒	☐	For hierarchical and complex designs, identification of the appropriate level for tests and full reporting of outcomes
☒	☐	Estimates of effect sizes (e.g. Cohen's d, Pearson's r), indicating how they were calculated

Our web collection on statistics for biologists contains articles on many of the points above.

Software and code

Policy information about availability of computer code

Data collection	For in vitro experiments,the cryo-SEM SUPRA 40VP-31-79 (Carl Zeiss SMT Ltd., Oberkochen, Germany) equipped with an EMITECH K250X cryopreparation unit (Quorum Technologies Ltd., Ashford, Kent, United Kingdom).EDS detector Bruker x-flash 6/100 and Esprit 2.0 Software (Bruker Corporation, Billerica, MA, USA). The 3D printer was controlled by the Repetier-Host software and the 3D models (STL-files) were converted to code (G-Code) by the software Slic3r (http://slic3r.org/). Confocal laser scanning microscope Zeiss LSM 780 (Carl Zeiss Microscopy GmbH, Jena, Germany) with the image exportation by ZEN 3.0 (Carl Zeiss Microscopy GmbH, Jena, Germany). For in vivo experiments, hydrogel volume and inguinal lymph node size were measured using dedicated 7T small animal magnetic resonance imaging (Bruker) and ParaVision 6.0.1. software. For volume determination of hydrogels with incorporated liquid metal particles the nanoScan® PET/CT (Mediso) with corresponding Nucline NanoScan software (3.00.020.0000) was used. Immunohistological stainings were imaged using AxioImager.A1 microscope and AxioVision software (version 4.8, Carl Zeiss).
Data analysis	For in vitro experiments, Unless otherwise noted, all experiments were performed in triplicate (n = 3) and data are presented as mean ± s.d. Statistical analysis was carried out using the software OriginPro 2017 (OriginLab Corp).For in vivo experiments, quantification of the hydrogel volume and lymphnode size was performed using the software ROVER (v3.0.57h ABX GmbH). Quantification of immunohistological stainings were performed using ImageJ/FIJI (version 1.52i). GraphPad Prism 7 and OriginPro 2017 was used for the generation of graphs and statistical data analysis.

For manuscripts utilizing custom algorithms or software that are central to the research but not yet described in published literature, software must be made available to editors and reviewers. We strongly encourage code deposition in a community repository (e.g. GitHub). See the Nature Research guidelines for submitting code & software for further information.

Data

Policy information about availability of data

All manuscripts must include a data availability statement. This statement should provide the following information, where applicable:

- Accession codes, unique identifiers, or web links for publicly available datasets
- A list of figures that have associated raw data
- A description of any restrictions on data availability

Data availability. All data supporting the results in this study are available within the article and its supplementary information or from the corresponding author upon reasonable request.

Field-specific reporting

Please select the one below that is the best fit for your research. If you are not sure, read the appropriate sections before making your selection.

☒ Life sciences ☐ Behavioural & social sciences ☐ Ecological, evolutionary & environmental sciences

For a reference copy of the document with all sections, see nature.com/documents/nr-reporting-summary-flat.pdf

Life sciences study design

All studies must disclose on these points even when the disclosure is negative.

Sample size	For in vitro experiments we did a preliminary experiment first, then set up the number of samples (n= 3-4) in different groups which based on the previous experience and standards in research (Adv Mater. 2018 May;30(22):e1706100 and Nat Commun. 2019 Jun 20;10(1):2705). In vivo experiments on female immunocompetent hairless SKH1-Elite mice were conducted to invesitigate in vivo biocompatibility based on the histological assessment after hydrogel injection, 3 mices for each group. The sample sizes of in vivoexperiments refers to previously published literature. (Advanced Functional Materials, 2017, 27(15): 1605189. and Biomaterials, 2014, 35(37): 9755-9766.)
Data exclusions	No data exclusions were made.
Replication	All the experiments were carried out in three replicates or more. Details of experimental replicates are given in the figure legends. All reported attempts atreplication were successful.
Randomization	All samples/organisms were randomly allocated into experimental group
Blinding	Regarding in vivo imaging and analysis, data sets and labeling contained only mouse numbers and no information on the injected materials or animal groups. The investigators were blinded to group allocation during data collection and analysis of immunohistological stainings. Blinding to group allocaiton was applied to all experiments during data collection.

Reporting for specific materials, systems and methods

We require information from authors about some types of materials, experimental systems and methods used in many studies. Here, indicate whether each material, system or method listed is relevant to your study. If you are not sure if a list item applies to your research, read the appropriate section before selecting a response.

Materials & experimental systems

n/a	Involved in the study	
☐	☒	Antibodies
☐	☒	Eukaryotic cell lines
☒	☐	Palaeontology and archaeology
☐	☒	Animals and other organisms
☒	☐	Human research participants
☒	☐	Clinical data
☒	☐	Dual use research of concern

Methods

n/a	Involved in the study	
☒	☐	ChIP-seq
☒	☐	Flow cytometry
☒	☐	MRI-based neuroimaging

Antibodies

Antibodies used	Primary antibody : anti-Cardiac Troponin T antibody (ab8295, abcam), anti-COX-2 (ab15191, Abcam), anti-TM (sc9162, Santa Cruz Biotechnology), anti-CD68 (MCA-1957, AbD Serotec), anti-TG-2 (sc20621, Santa Cruz Biotechnology), anti-VEGF (sc152, Santa Cruz Biotechnology),anti-CD31 (ab28364, Abcam). normal rat IgG (sc-2026, Santa Cruz Biotechnology).<br><br>Secondary antibody: Donkey anti-Mouse IgG (H+L) ReadyProbes™ Secondary Antibody, Alexa Fluor 488 (life technologies, R37114) IgG-biotinylated (111-065-003, Dianova),IgG-biotinylated (312-066-045, Dianova). anti-mouse Alexa Fluor™ 647 (Thermo Fisher,

	Invitrogen™, C10340).
Validation	Each antibody was validated for the species (mouse or rabbit) and application IF by the correspondent manufactur.

Eukaryotic cell lines

Policy information about cell lines

Cell line source(s)	C2C12 cell from ECACC, L929 cells from ECACC.
Authentication	C2C12 and L929 cell lines were authenticated by ECACC. STR-based profiling method was used by ECACC.
Mycoplasma contamination	The cell lines were not tested for mycoplasma contamination.
Commonly misidentified lines (See ICLAC register)	No cell lines used in this study were found in the database of commonly misidentified cell lines that is maintained by ICLAC and NCBI Biosample.

Animals and other organisms

Policy information about studies involving animals; ARRIVE guidelines recommended for reporting animal research

Laboratory animals	female immunocompetent hairless SKH1-Elite mice (age 8-9 weeks, weight 20-25 g) were purchased from Charles River laboratories.
Wild animals	This study does not involve wild animals.
Field-collected samples	This study does not involve field-collected animals.
Ethics oversight	Animal experiments were conducted in accordance with the guidelines of German Regulations for Animal Welfare. The local Ethical Committee for Animal Experiments ("Landesdirektion Sachsen") approved the underlying protocol (reference number DD24.1-5131/450/16) and the animal experiments were conducted at the Helmholtz-Zentrum Dresden-Rossendorf (Institute of Radiopharmaceutical Cancer Research).

Note that full information on the approval of the study protocol must also be provided in the manuscript.
